# Epigenetic machinery is functionally conserved in cephalopods

**DOI:** 10.1186/s12915-022-01404-1

**Published:** 2022-09-14

**Authors:** Filippo Macchi, Eric Edsinger, Kirsten C. Sadler

**Affiliations:** 1 Program in Biology, New York University Abu Dhabi, P.O. Box 129188, Abu Dhabi, United Arab Emirates; 2 Molecular Neurobiology Laboratory, The Salk Institute for Biological Studies, 10010 Torrey Pines Road, La Jolla, CA 92037 USA

**Keywords:** *Octopus bimaculoides*, Cephalopods, Epigenetics, DNA methylation, Histone methylation, DNMT1, UHRF1

## Abstract

**Background:**

Epigenetic regulatory mechanisms are divergent across the animal kingdom, yet these mechanisms are not well studied in non-model organisms. Unique features of cephalopods make them attractive for investigating behavioral, sensory, developmental, and regenerative processes, and recent studies have elucidated novel features of genome organization and gene and transposon regulation in these animals. However, it is not known how epigenetics regulates these interesting cephalopod features. We combined bioinformatic and molecular analysis of *Octopus bimaculoides* to investigate the presence and pattern of DNA methylation and examined the presence of DNA methylation and 3 histone post-translational modifications across tissues of three cephalopod species.

**Results:**

We report a dynamic expression profile of the genes encoding conserved epigenetic regulators, including DNA methylation maintenance factors in octopus tissues. Levels of 5-methyl-cytosine in multiple tissues of octopus, squid, and bobtail squid were lower compared to vertebrates. Whole genome bisulfite sequencing of two regions of the brain and reduced representation bisulfite sequencing from a hatchling of *O. bimaculoides* revealed that less than 10% of CpGs are methylated in all samples, with a distinct pattern of 5-methyl-cytosine genome distribution characterized by enrichment in the bodies of a subset of 14,000 genes and absence from transposons. Hypermethylated genes have distinct functions and, strikingly, many showed similar expression levels across tissues while hypomethylated genes were silenced or expressed at low levels. Histone marks H3K27me3, H3K9me3, and H3K4me3 were detected at different levels across tissues of all species.

**Conclusions:**

Our results show that the DNA methylation and histone modification epigenetic machinery is conserved in cephalopods, and that, in octopus, 5-methyl-cytosine does not decorate transposable elements, but is enriched on the gene bodies of highly expressed genes and could cooperate with the histone code to regulate tissue-specific gene expression.

**Supplementary Information:**

The online version contains supplementary material available at 10.1186/s12915-022-01404-1.

## Background

Epigenetic modifications to histones and DNA regulate tissue-specific profiles of gene expression, repress transposable elements (TEs), and organize the genome into euchromatin and heterochromatin domains [1]. There is great diversity across the animal kingdom in how the epigenome accomplishes these complex and important functions [2]. Elucidating the mechanisms of epigenome patterning, regulation, and function are important to understand how epigenetic marks regulate genes and TEs and orchestrate genome organization across the evolutionary tree.

Examining DNA methylation in diverse animal species provides an illustrative example of how incorporating organism diversity into epigenetic studies expands the epigenetic lexicon [3]. In vertebrates, the vast majority of CpGs are methylated, with an asymmetric distribution throughout the genome. 5-Methyl-cytosine (5mC) is enriched in intergenic regions, excluded from CpG-rich promoters, and is present in a mosaic pattern on gene bodies, with some genes characterized as hypermethylated and others lacking methylation [4, 5]. Extensive methylation of repetitive sequences reflects its important function in suppressing the expression of transposons [6]. In contrast, the initial studies on canonical invertebrate model organisms—*Caenorhabditis elegans* and *Drosophila melanogaster*—showed that these animals were devoid of DNA methylation [7–9], whereas less commonly used models such as tunicates [10] and sea urchins have CpG methylation but at levels considerably lower than vertebrates [8, 11]. As functional analysis of DNA methylation in animals has been primarily carried out using vertebrate model organisms and human samples, expanding the field of comparative epigenomics to decipher the function of DNA methylation in other species is an important goal.

The advance of sequencing technologies has massively expanded the diversity of organisms with fully sequenced genomes, allowing examination of DNA methylation patterns in animals across the evolutionary tree [3, 12–17]. Such studies confirm early observations that most invertebrates have either low levels of CpG methylation or lack DNA methylation entirely and that the pattern of 5mC distribution is very different in animals with low levels of methylation compared to hypermethylated vertebrate genomes. In vertebrates, repetitive elements and some gene bodies are highly methylated, while CpG islands in promoters are protected from methylation [12–15, 18]. In animals characterized by low DNA methylation levels (i.e., <20% of CpGs methylated), DNA methylation is enriched in some gene bodies, with heavily methylated genes on average expressed at higher levels than unmethylated genes [3, 5, 12, 13, 19–22]. Exceptions to these patterns abound; for example, the methylome pattern in the sponge *Amphimedon queenslandica* is highly similar to vertebrates [23], the annelid *Platynereis dumerilii* [24] has a high level of DNA methylation in the larval stages that decreases in juveniles and then increases again when the animals achieve sexual maturation, sea squirts show a high level of gene body methylation with an intermediate methylation pattern on repeats [10, 13, 14, 17], and dynamic changes in the DNA methylation pattern during oyster development [21, 25] have been observed. Therefore, the pattern of 5mC in the genome can vary even among animals who have equivalent levels of total DNA methylation, potentially reflecting divergent functions of cytosine methylation in different contexts.

Cephalopod genomes have unique organization and structural features [26–29]. Two recent reports highlighted some of these novelties in genome organization and transcriptional regulation in *Octopus bimaculoides*, the Boston market squid, *Doryteuthis pealeii*, and the bobtail squid, *Euprymna scolopes*, including clustering of genes into distinct genomic domains called microsyntenies and a massive restructuring of the genome compared to closely related animals [26, 27]. Moreover, features of the *O. bimaculoides* methylome was highlighted by a recent study using whole genome bisulfite sequencing (WGBS) to analyze brain methylation patterns in diverse animal species [22]*.* In most animals, TE abundance correlates with genome size, and since DNA methylation serves to keep transposons in check in vertebrates and plants [5], it has been hypothesized that DNA methylation is a key to suppressing TE expression in organisms with large genomes and high transposon burden [30]. This is not the case in *O. bimaculoides*, where half the genome is populated by repetitive elements similarly to vertebrates [29]. Interestingly, the level of DNA methylation is not proportional to the transposon load in this genome [22], and there is a high level of TE expression in octopus tissues, especially in the brain [26, 29, 31]. This warrants investigation of the function of DNA methylation in these animals.

In species with methylated genomes, the pattern of CpG methylation is maintained between parent and daughter cells by the DNA methyltransferase 1 (DNMT1) which is targeted to hemi-methylated DNA generated during DNA replication by the Ubiquitin Like With PHD And Ring Finger Domains 1 (UHRF1) protein [32–37]. UHRF1 also functions as a reader of the histone code, including the canonical heterochromatin mark, trimethylated histone H3 lysine 9 (H3K9me3) [38–43]. Thus, the DNMT1-UHRF1-complex represents the core DNA methylation machinery and contributes to establishing heterochromatin domains. Recent studies reported high conservation of DNMTs, UHRF1, and TET proteins across diverse phylogenetic groups of animals including annelid worms [24], sponges [23, 24], mollusks [20, 44], and other invertebrates [3, 22], and even in a fungus [45]. This suggests that although 5mC is not ubiquitous throughout the animal kingdom, in cases where it is present, it is mediated by the same complexes that function in vertebrates.

Cephalopods represent an emerging model system with multiple studies utilizing squid, bobtail squid, and octopus for uncovering novel mechanisms of RNA editing, highly complex behavioral regulation, remarkable regenerative capacity, and genome evolution [26, 27, 46–54]. Recent advances in embryo cultivation, standardized aquaculture protocols [55], and genome editing [56] have advanced the utility of cephalopods as new model organisms [28]. Transcriptomic profiling of mollusk embryos [57], brain [58, 59], and multiple tissues of *O. bimaculoides* adults [29] has revealed that key development and neurological processing genes are highly conserved in these animals. However, a comprehensive analysis of tissue-specific gene expression profiles in cephalopods has not been reported. Moreover, despite significant strides in cephalopod research and few studies reporting the presence of DNA methylation in some species of octopus [22, 60, 61], there is virtually nothing known about the epigenetic marks that contribute to the regulation of tissue-specific gene expression profiles, transposon suppression, or genome organization in cephalopods.

We address this using transcriptomic and methylome datasets and biochemical approaches to analyze *O. bimaculoides* tissue-specific gene expression, methylation patterning, and histone modifications. Using biochemical analysis, we show that DNA methylation and histone modifications are present in multiple tissues of three cephalopod species (*O. bimaculoides*, *D. pealeii*, *E. berryi*). Our methylome analysis in octopus shows that methylated CpGs account for less than 10% of all CpGs in the genome and are virtually absent from repetitive DNA and transposons. Methylated CpGs are clustered on the gene bodies of a distinct set of genes, which are highly expressed across tissues. This shows that CpG methylation and histone methylation are prominent features of the cephalopod epigenome and suggests that the pattern of DNA methylation is set by characteristics of the genome that are maintained across cell types.

## Results

### Expression profile analysis identifies tissue-specific gene clusters in octopus

**Fig. 1:**
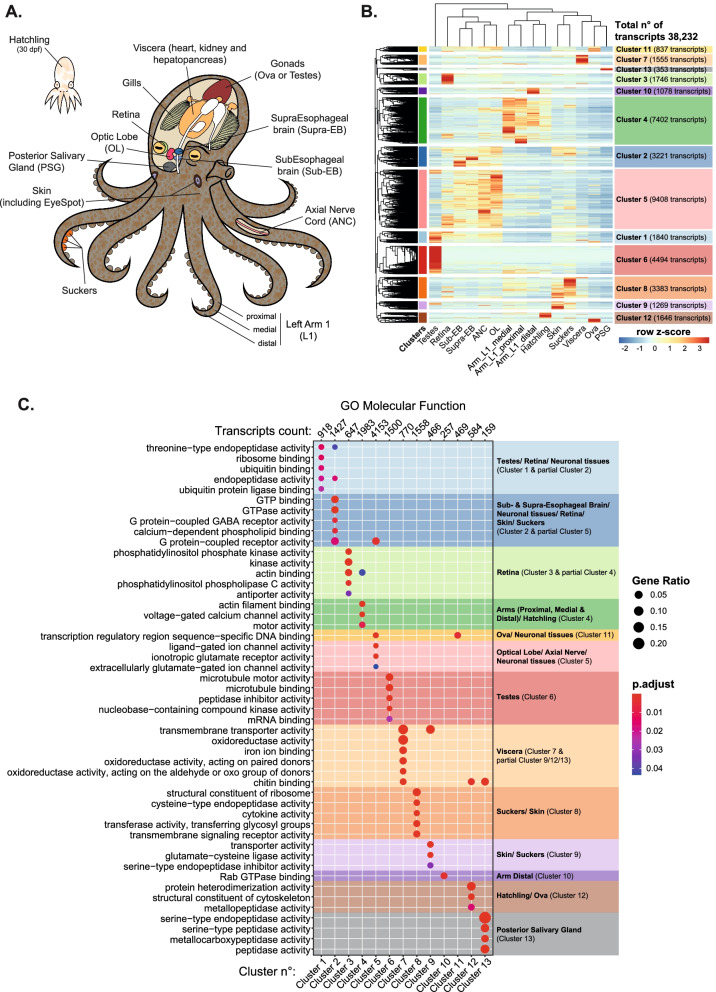
Expression profiling identifies tissue-specific gene clusters with distinct functional annotation. **A** Schematic representation of *O. bimaculoides* anatomy highlighting the tissues analyzed in this study. **B** Heatmap of the expression profile of 13 different tissues (including 12 different tissues derived from adult animals and one whole-body hatchling) with *z*-scores based on rows. Rows are divided into 13 clusters calculated on the hierarchical clustering of dendrogram (Euclidean distance). **C** Gene Ontology (GO) of genes in each cluster. Dot size represents gene ratio between observed and expected transcripts in each GO category (with padj < 0.05), dot colors represent adjusted *p*-value

We extended previously published RNA-seq datasets obtained from 11 different adult tissues from males, females, and 30 dpf hatchlings (Fig. 1A; [27, 29]) by generating new RNA-seq datasets from the first left (L1) arm of an adult male octopus at distal, medial, and proximal locations (between 1.5 and 5.5 cm from the arm tip, Additional file 1: Fig. S1A-B), and one whole-body 30-day-old hatchling (Additional file 1: Fig. S1C-D, Additional file 2: Table S1). We combined these datasets and found that of 40,327 transcripts annotated in the *O. bimaculoides* genome, 38,232 were expressed in at least one sample (TPM > 0). Trinotate [62] was used to better annotate the *O. bimaculoides* genome and assigned putative gene names and putative UniProt entry names to 23,879 transcripts (59%) identified in the datasets analyzed (Additional file 3: Table S2).

Optimized hierarchical clustering of the 38,232 expressed transcripts generated 13 clusters that define specific transcriptomic profiles across these tissues (Fig. 1B and Additional file 4: Table S3). Some clusters were dominated by transcripts that were nearly exclusively expressed in a single tissue, such as Cluster 6 (testes), whereas other clusters were defined by transcripts expressed in multiple tissues that are functionally related, such as Cluster 5 (tissues with neuronal functions). Interestingly, the distal arm segment was characterized by a distinct set of transcripts in Cluster 10 that were not highly enriched in any other tissue (Fig. 1B), potentially reflecting the sensory and regenerative capacity of this structure [46, 51, 63].

To determine whether the sex of the animal could influence the tissue-specific expression profile, we compared the same tissue from 2 male and 1 female adult octopus. We compared the transcriptome from the distal tip of arm L1 (1.5 cm from the tip) derived from the male (OB-5) used for the hierarchical clustering in Fig. 1B to the arm tip obtained from an additional male (OB-2) and female (OB-9, Additional file 2: Table S1). The top 1000 expressed transcripts in each sample were combined in a unified set of 1405 genes, with nearly half (622 transcripts) common to all samples (Additional file 5: Fig. S2A) and with the greatest similarity between the female and male arm tips (Additional file 5: Fig. S2B) compared to the more distal region of another male. This suggests that sex dimorphism does not generate major differences in clustering analysis of tissues that appear similar between males and females.

Gene Ontology (GO) analysis for Molecular Function (Fig. 1C), Biological Process (Additional file 6: Fig. S3A), and Cellular Component (Additional file 6: Fig. S3B) revealed that each cluster was enriched for transcripts encoding proteins with shared functions. For example, microtubule binding, motor activity, and cilium movement, terms which are prominent for sperm activity, characterized genes expressed in testes (i.e., Cluster 6; Fig. 1C and Additional file 6: Fig. S3). G-protein coupled receptor activity, GTP binding, glutamate receptor activity, and signaling pathways and others critical for neuronal function were enriched in Clusters 2 and 5 (brain and nervous tissues; Fig. 1C and Additional file 6: Fig. S3), as found by others [27, 29]. Cluster 4, which was enriched in transcripts expressed in the arm and hatchling, included terms involved in motor activity, actin filament binding, calcium-ion transmembrane transport, and myosin complex (Fig. 1C and Additional file 6: Fig. S3), reflecting contractile activities of muscles. This analysis provides tissue-specific functionally annotated genesets for octopus.

### DNA methylation machinery is conserved and differentially expressed in octopus

**Fig. 2:**
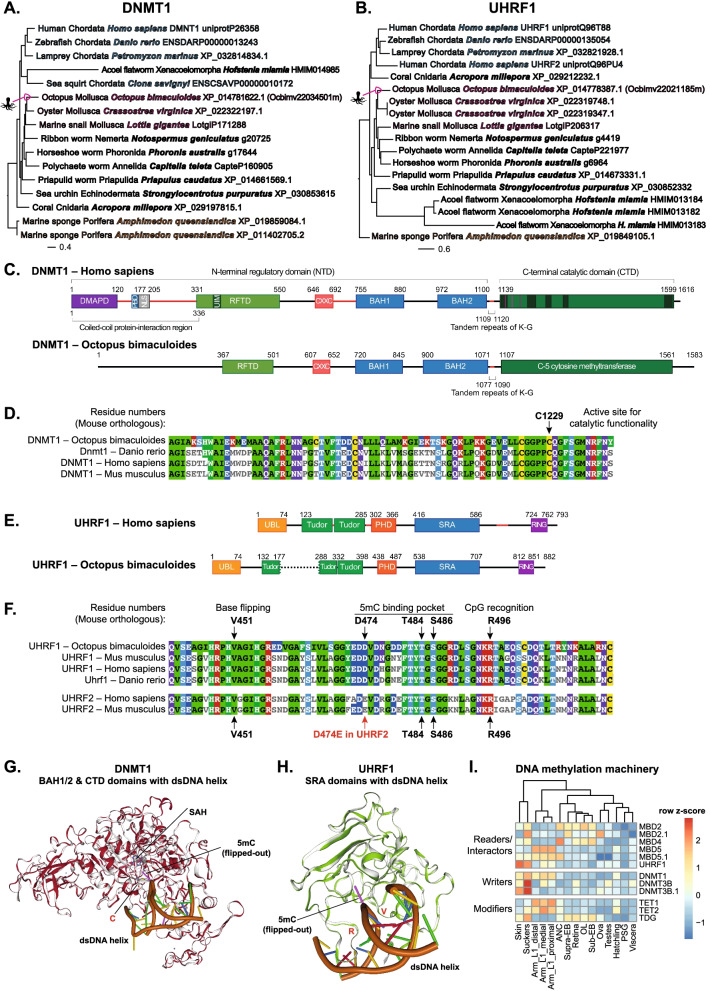
DNA methylation machinery is conserved and differentially expressed across different tissues. Phylogenetic trees of **A** DNMT1 and **B** UHRF1 in a representative subset of 19 metazoan and outgroup species. Colors indicate phyla (blue = Chordata; pink = Mollusca; orange = Porifera), and octopus are indicated with an icon. **C** DNMT1 domain structure in *H. sapiens* and *O. bimaculoides*. Numbers indicate amino acid residues for each species. **D** Alignment of the C-terminal Catalytic Domain (CTD) of *O. bimaculoides* to *M. musculus*, *H. sapiens*, and *D. rerio* shows that the major residue needed for DNMT1 catalytic function is highly conserved among the species. Residue functionality was assigned based on the mouse DMNT1 ortholog. **E** Domain structure of UHRF1 in *H. sapiens* and *O. bimaculoides*. **F** Alignment of the *O. bimaculoides* SRA domain to *M. musculus*, *H. sapiens*, and *D. rerio* shows that all major residues needed for UHRF1 functionality are conserved among the species. Residue functionality is based on the mouse UHRF1 ortholog. Alignment to UHRF2 shows no conservation of the critical residues between the SRA domain in *O. bimaculoides* and UHRF2 in *H. sapiens* and *M. musculus*. **G** Structural superposition of the 3D structure of BAH1, BAH2, and CTD domains in *M. musculus* (grey) with the 3D model of the same domains in *O. bimaculoides* (red). DNA is represented in brown, and critical residues for 5mC deposition are highlighted in red. **H** Structural superposition of the 3D structure of the SRA domain in *M. musculus* (grey) with the 3D model of the same domain in *O. bimaculoides* (green). DNA is represented in brown and residues critical for CpGs recognition and 5mC base flipping are highlighted in red. **I** Expression profiles of DNA methylation machinery. Gene names are extracted from Trinotate (Table S6)

A recent finding of a low level of DNA methylation in octopus compared to vertebrates [22] led us to investigate the DNMT and UHRF gene family evolution in animals using a phylogenomic pipeline we designed [64]. Human DNMT1, TRDMT1 (DNMT2), DNMT3A, DNMT3B, and DNMT3L and human UHRF1 and UHRF2 were compared against Metazoa19 and Metazoa50 genomes (Additional file 7: Table S4). We identified DNMT1 (Ensembl Transcript ID: Ocbimv22034501m; NCBI gene symbol: LOC106877272; Fig. 2A and Additional file 8: Fig. S4A) and UHRF1 (Ensembl Transcript ID: Ocbimv22021185m; NCBI gene symbol: LOC106874972; Fig. 2B and Additional file 8: Fig. S4B) homologs in *O. bimaculoides* and across a range of animal species*.* Both DNMT1 and UHRF1 are absent in the three unicellular outgroups examined (choanoflagellate, *Monosiga brevicollis*, the flilasterean, *Capsaspora owczarzaki* and the ichthyosporean, *Sphaeroforma arctica*), but highly conserved in most animals and were likely present in the last common ancestor, with a number of independent losses in diverse lineages. DNMT1 was lost in 11 out of 47 Metazoa50 animal species, including the fruit fly *Drosophila melanogaster* (but was present in two other arthropods), and nematodes. UHRF losses were detected for 12 out of 47 Metazoa50 animal species, which match losses of DNMT1 except for the sea squirt, *Ciona savignyi*, which is predicted to have low level of DNA methylation [65]. Several species or clades also exhibited expansions to two or three copies for both genes (Fig. 2A, B and Additional file 8: Fig. S4A-B).

The DNMT1 transcript (hereafter termed DNMT1_OCTBM) encodes a protein that retains the domain structure (Fig. 2C) and key amino acid residues (Fig. 2D) that are necessary for its methyltransferase function. HMMER analysis of DNMT1_OCTBM to identify sequence homology in the animal reference protein database showed that the C-5 cytosine methyltransferase domain (Pfam ID: DNA_methylase), which carries out the catalytic function of DNMT1, had the highest homology score and, overall, DNMT1_OCTBM had over 50% of identity with vertebrate DNMT1s (Additional file 9: Fig. S5A-B). We conclude that DNMT1_OCTBM has the necessary features to function as a DNA methyltransferase.

There are UHRF1 and UHRF2 family members in mammals, but there is only 1 family member present in mollusks (Additional file 8: Fig. S4B and [24]). The protein encoded by Ocbimv22021185m (hereafter termed UHRF1_OCTBM) retains features of the mammalian protein, including tandem Tudor and PHD domains that read the histone code and the SRA domain, which binds hemi-methylated DNA and facilitates DNMT1 access to CpGs (Fig. 2E) [32–35, 39, 40, 66, 67]. The SRA domain of UHRF1_OCTBM has highest homology compared to vertebrate homologs (Additional file 9: Fig. S5C-D), with key residues involved in hemi-methylated DNA recognition and base flipping [33, 35] completely conserved (Fig. 2F). There was much lower homology between UHRF1_OCTBM and UHRF2, and the 5mC binding pocket (residue D474E), the base flipping motif (HVAG thumb loop), and the CpG recognition site (NKRT finger loop) [34] were not conserved (Fig. 2F). Another octopus transcript (Ocbimv22020196m; termed YDG-OCTBM), encoded a shorter protein containing a domain similar to the SRA but had low homology to vertebrate UHRF1 or UHRF2 (Additional file 9: Fig. S5E-F) and lacked key residues for CpG recognition found in both UHRF1 and UHRF2 (Additional file 9: Fig. S5G). The conservation protein structure was demonstrated using Swiss-Expasy 3D modeling [68], which revealed highly conserved 3D structures for DNMT1_OCTBM compared to the mouse protein (Fig. 2G, Additional file 10: Fig. S6A-B), with the same positioning of the DNA double helix, 5mC, and S-Adenosyl-L-methionine (Fig. 2G, Additional file 10: Fig. S6A-B). The structure of the SRA domain of UHRF1_OCTBM protein is very similar to mouse protein, with the loops necessary to bind DNA, recognize CpGs (R612 and R496 in mouse [33, 35]), and mediate base flipping (V567 and V451 in mouse) all properly positioned (Fig. 2H, Additional file 10: Fig. S6C-D). Thus, Ocbimv22034501m encodes the closest DNMT1 homolog and Ocbimv22021185m encodes the UHRF1 ortholog in *O. bimaculoides* and the octopus proteins retain all the properties needed to interact as a complex, for UHRF1 to recognize hemi-methylated DNA and for DNMT1 to methylated CpGs. We conclude that these proteins function as the DNA methylation machinery in octopus.

Since UHRF1 and DNMT1 function as a complex during S-phase, they are typically co-expressed in cells that are actively proliferating. We found low expression levels of both of these genes in most tissues, with the highest levels in skin, suckers, and arms (Fig. 2I, Additional file 11: Table S5, Additional file 12: Table S6), which are tissues that undergo high turnover [63]. We also show that most genes encoding factors that read (methyl binding proteins; MBD), write (DNMTs), and erase (ten-eleven translocation; TET) DNA methylation were also expressed at low levels in most tissues in octopus, except for arms, suckers, and skin (Fig. 2I, Additional file 11: Table S5, Additional file 12: Table S6), suggesting that DNA methylation remodeling may be most prominent in these tissues.

### DNA methylation is enriched in the bodies of a subset of genes in *O. bimaculoides*

**Fig. 3:**
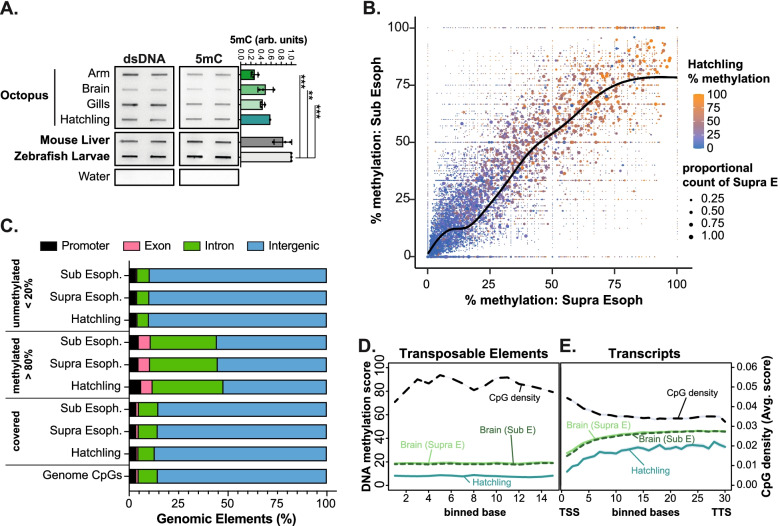
DNA methylation is enriched in a subset of gene bodies in *O. bimaculoides*. **A** Slot blot of gDNA extracted from the distal arm tip (1.5 cm of right arm 2), brain (optical lobe), and gills of one representative *O. bimaculoides* adult animal, and whole 30-day-old hatchling (same biological sample used for RNA-seq and RRBS). gDNA extracted from one representative male mouse adult liver and one representative pool of whole 5 dpf zebrafish larvae was blotted on the same membrane for comparison. Water was used as negative control. The 5mC signal was normalized to double-stranded DNA (dsDNA), and then each sample was normalized to levels zebrafish larvae. Each dot represents one biological replicate, bars represent the mean among biological replicates, and error bars represent standard deviations. *p*-values were calculated by unpaired parametric one-way ANOVA test adjusted with Tukey’s multiple comparisons test. Adjusted *p*-value are indicated as *** < 0.001, ** < 0.01. Comparisons other than with zebrafish or mouse resulted in not significant adjusted *p*-values > 0.05 (not indicated in the graph). **B** Scatter plot of DNA methylation levels of common CpGs across Supra Esophageal (Supra E) and Sub Esophageal (Sub E) brain. Dot color represents DNA methylation levels in hatchling, and size of the dots indicates scaled proportion of CpGs represented by each dot. **C** Genomic annotation of CpGs contained in *O. bimaculoides* genome, and of those covered by WGBS in brain and RRBS in hatchling. CpGs were divided into methylated (> 80%) and not methylated (< 20%) CpGs and relative genomic annotation was performed. **D** Metaplot represents DNA methylation levels of transposable elements in Supra E and Sub E brain and hatchling. CpG density of octopus genome is represented for the same region. Each region is divided in 15 bins. **E** Metaplot represents the DNA methylation levels in Supra E and Sub E brain and hatchling for full-length transcripts. CpG density of octopus genome has been represented for the same region. Each transcript has been divided in 30 bins from transcription star site (TSS) to transcription termination site (TTS)

Reports of the presence of DNA methylation in octopus [22, 60, 61] and other mollusks [19, 21, 25] indicate that the DNA methylation machinery is functional in these animals. Slot blot analysis to detect total double-stranded DNA and bulk 5mC levels on genomic DNA (gDNA) extracted from arm tip, brain (optic lobe), and gills of 3 different *O. bimaculoides* adults and a hatchling (Additional file 2: Table S1) showed relatively equivalent levels in all tissues, albeit at less than half of what was detected in mouse liver or zebrafish larvae. As a negative control, samples lacking any DNA were devoid of signal with both antibodies (Fig. 3A, Additional file 13: Fig. S7).

We next examined the genome-wide distribution of methylated and unmethylated CpGs by performing RRBS on gDNA isolated from a 30 dpf hatchling and by analyzing previously published WGBS datasets from 2 regions of the adult brain (Supra Esophageal - Supra E; and Sub Esophageal - Sub E) [22]. There are 80,660,576 CpGs in the *O. bimaculoides* genome, considering both DNA strands. RRBS profiling of the hatchling genome covered 2.98% of these (2,403,266 CpGs) and WGBS of the Supra E and Sub E covered 80.39% (64,845,686 CpGs) and 58.33% (47,053,504 CpGs), respectively (Additional file 14: Fig. S8A). In all samples, the vast majority of CpGs had methylation levels categorized as not methylated (i.e., methylated in <20% of reads). Very few CpGs were categorized as methylated (i.e., methylated in >80% of reads), ranging from 3.63% of CpGs in hatchlings and 7.53% in the Sub E brain (Additional file 14: Fig. S8A-B), with the differences between these potentially due to differences in sequencing depth and method of methylome profiling. Approximately 7% of CpGs in all samples showed an intermediate pattern of methylation (between 20 and 80%) which could reflect tissue heterogeneity or a stochastic pattern of methylation on these CpGs. This methylation pattern (Additional file 14: Fig. S8B) contrasts the bimodal distribution found in tissues from vertebrates and some species of sponge [23, 24], where the majority of CpGs are methylated, and the remaining are unmethylated, but very few have an intermediate methylation level. These findings indicate that the octopus methylome resembles other invertebrates [3, 5, 12, 17], including another mollusk, the Japanese oyster *Crassostrea gigas* [3, 20], but is distinct from invertebrate model organisms, which lack methylation entirely.

DNA methylation serves to repress transposon expression in vertebrates, and therefore the bulk of methylated CpGs are found in intergenic regions, with very little tissue-specific variation [3, 69, 70]. To investigate if the methylation pattern varied across octopus tissues, we identified 1,425,557 CpGs that were commonly detected in the brain and hatchling methylome datasets, and then plotted the methylation levels on each CpG in the two brain samples and overlaid the methylation levels from the hatchling (Fig. 3B). This showed a linear correlation between brain samples and that nearly all CpGs that were either unmethylated or highly methylated in the brain samples had the same pattern in hatchling (Fig. 3B). Pairwise comparison of hatchling to SupraE (Additional file 14: Fig. S8C) and hatchling to SubE (Additional file 14: Fig. S8D) showed a similar linear correlation. Analysis of a broader range of tissues will be useful to determine if this similar methylation pattern is maintained in other octopus tissues.

The vast majority of the *O. bimaculoides* genome is intergenic and nearly half of the genome is occupied by repetitive sequences [26, 29, 71]. Over 85% of CpGs in the octopus genome were intergenic (Fig. 3C). These CpGs are comparatively depleted of methylation, and there is a strong enrichment of methylated CpGs (>80%) detected in introns and exons (Fig. 3C). Moreover, while there is high CpG density, on average, across the length of TEs, methylation levels were consistently low on all TE classes compared to genes (Fig. 3D, Additional file 15: Fig. S9A-B). Interestingly, there was a relatively higher level of methylation in satellite repeats (Additional file 15: Fig. S9A), even when the number of CpGs was randomly down-sampled to match the number of CpGs found in satellites (Additional file 15: Fig. S9B). To better investigate DNA methylation of repetitive elements, we selected CpGs present in transposable elements and other repetitive elements in intergenic regions, comparing their methylation levels to intergenic regions that did not contain repetitive elements. This showed that repetitive elements had higher levels of methylation compared to intergenic regions that lacked repeats, suggesting that although DNA methylation is relatively low on TEs, it is higher than in regions that lack TEs (Additional file 15: Fig. S9C-D). Reports of high levels of TE expression in the octopus brain suggest a complex method of their regulation [27, 29, 31] and although DNA methylation may play a role in regulating some TEs family, the low level of DNA methylation on transposons suggests it is not a major epigenetic mechanism of TE repression in octopus.

**Fig. 4:**
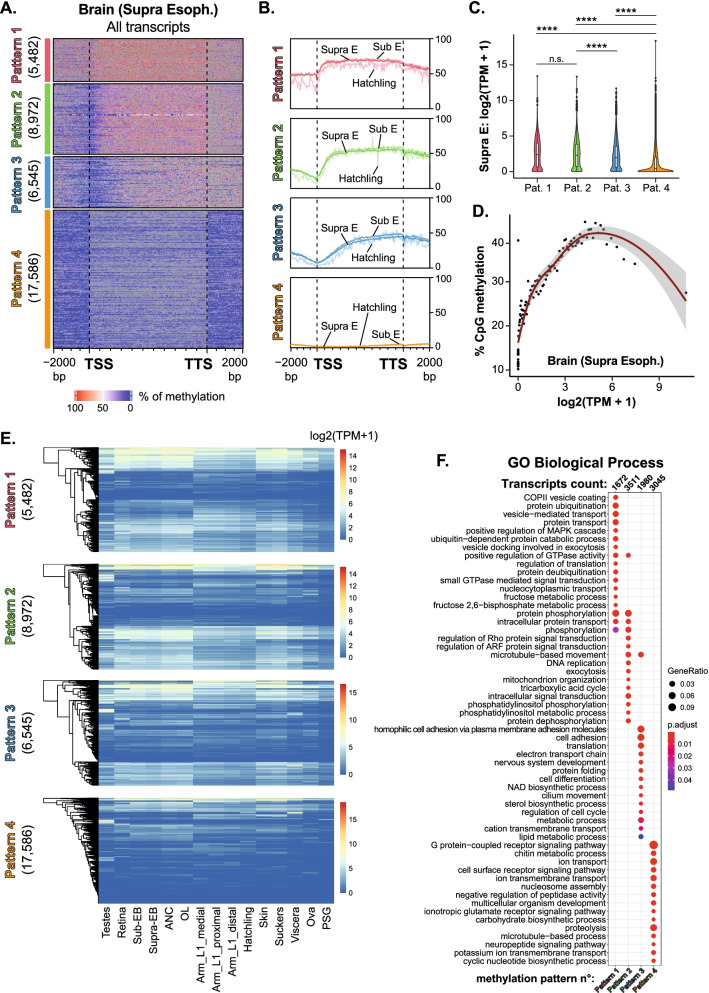
DNA methylation on gene bodies correlates with gene expression. **A** Heatmap of DNA methylation levels detected in Supra E brain samples across full-length transcripts and 2000 bp upstream and downstream of start site. All transcripts have been divided in 4 clusters by k-means based on the DNA methylation average across each transcript. **B** Line plot represents average of DNA methylation of Supra E and Sub E brain and hatchling for each cluster defined in the heatmap on Supra E sample. **C** Violin plot displays the distribution of transcript expression values (as log2(TPM+1)) for each methylation pattern in Supra E brain samples. Box-and-whisker plots inside the violin have a center line at the median, lower and upper hinges correspond to first and third quartiles, and whiskers extend from hinges to largest or smallest values no further than 1.5 × IQR (inter-quartile range), while data beyond the end of the whiskers are outlying points that are plotted individually. *p*-values were calculated by unpaired non-parametric Kruskal-Wallis test adjusted with Dunn’s multiple comparisons test. **** indicates adjusted *p*-value < 0.0001, and n.s. indicates not significant adjusted *p*-values > 0.9999. **D** Overall DNA methylation of transcripts (TSS to TTS of each transcript) in Supra E brain is regressed against transcripts expression (log2(TPM+1)). Transcripts were grouped by percentile of expression values and each dot represents the average value of DNA methylation for each percentile. **E** The expression profile (as log2(TPM+1)) of transcripts categorized in each methylation pattern have been represented across the 13 different tissues. Column clustering is supervised according to the sample order established in Fig. 1B. Row clustering is calculated on the hierarchical clustering of dendrogram (Euclidean distance). **F** Gene Ontology (GO Biological Process) annotation of transcripts in each methylation pattern. Dot size represents gene ratio between observed and expected transcripts in each GO category (with padj < 0.05), dot colors represent adjusted *p*-value

In contrast to TEs and intergenic sequences, CpG methylation was comparatively higher in gene bodies, but the average methylation level across gene bodies did not exceed 50% in any tissue (Fig. 3E). We hypothesized that in cephalopods, like in other species with low DNA methylation levels, gene bodies are the main target of methylation [5]. The 5mC is a binary mark, so that each residue in individual cells can be either methylated or unmethylated. Therefore, we reasoned that the intermediate methylation level observed across all gene bodies represented heterogeneity in the CpG methylation pattern of genes. We tested this in the Supra E brain sample since it was the sample with the highest amount of covered CpGs (Additional file 14: Fig. S8A). K-means clustering of genes based on CpG methylation levels averaged for each transcript identified 4 distinct Methylation Patterns (MP; Fig. 4A, B): MP 1 had the fewest number of genes (5482) and was characterized by having high CpG methylation across the entire gene body and promoter; this was distinguished from genes in MP 2 (8972), which had high methylation across the gene body and downstream of the transcription termination site (TTS) but lacked methylation at the transcript start site (TSS) and promoter. Genes in MP 3 (6545) were characterized by having unmethylated CpGs in the first third of the gene body but higher methylation in the 3′ end of the gene and around the TTS. MP 4 was the inverse of MP 1, with the largest number of genes (17,586) lacking methylated CpGs (Fig. 4A and Additional file 16: Table S7). Strikingly, the same MPs were detected in the Sub E and hatchling samples (Fig. 4B, Additional file 17: Fig. S10A-B). This indicates that the pattern of DNA methylation on a gene is set by a cellular or genomic feature that is consistent across tissues.

We determine the relationship between gene body methylation and expression by plotting the expression (log2(TPM+1)) of all the transcripts in each MP. The highly methylated genes (MP 1-2) were expressed at the highest levels, while unmethylated genes (MP 4) were expressed at the lowest levels in the Supra E samples (Fig. 4C). To examine the direct correlation between methylation level and expression, transcripts in the Supra E sample were grouped into percentiles based on expression levels (log2(TPM+1)) and then the mean methylation level for all genes in each percentile (Fig. 4D) was calculated. This revealed a direct correlation between DNA methylation and expression for genes at the mid-range of expression levels (1.5 to 4.5 log2(TPM+1)). However, there was minimal or no correlation between methylation levels and expression in unmethylated genes (Fig. 4D). This relationship was also found in Sub E and hatchling samples (Additional file 17: Fig. S10C). Strikingly, a subset of genes in MP 1 and 2 were expressed at high levels in all tissues, whereas genes in MP 4 were either silenced or expressed at more variable levels across tissues (Fig. 4E). These data, showing the positive correlation between gene body methylation and constitutive expression, suggest either that gene body methylation sustains gene expression in octopus, as found in other animals [12, 13, 16, 20], or that DNA methylation in gene bodies is only a passenger of gene transcription.

GO analysis showed that specific gene functions were enriched in each MP. Trafficking, signaling, translation, and metabolism characterized genes in MP 1-2 and the unmethylated genes in MP 4 participated in metabolism, processes that occur at the cell surface, or are related to neuronal functions (Fig. 4F). Thus, the pattern of CpG methylation on gene bodies, or lack thereof, defines genes that play roles in similar biological processes and that genes in MP 1 may be involved in processes that are required by all cells, such as protein secretion, metabolism, and translation.

Studies in other invertebrates suggested a correlation between gene body methylation, gene length, and age [20]. Transcripts in each *O. bimaculoides* MP had a large range of average lengths (from TSS to TTS), with the highest variability in fully methylated transcripts (MP 1). The average length of unmethylated genes (i.e., in MP 4) was shorter than genes that were methylated (Additional file 17: Fig. S10D). In summary, this analysis shows that there is a similar pattern of DNA methylation on genes across tissues that positively correlate with gene expression and that genes with distinct functions are marked by similar methylation patterns.

### Histone-modifying enzymes are conserved in octopus

**Fig. 5:**
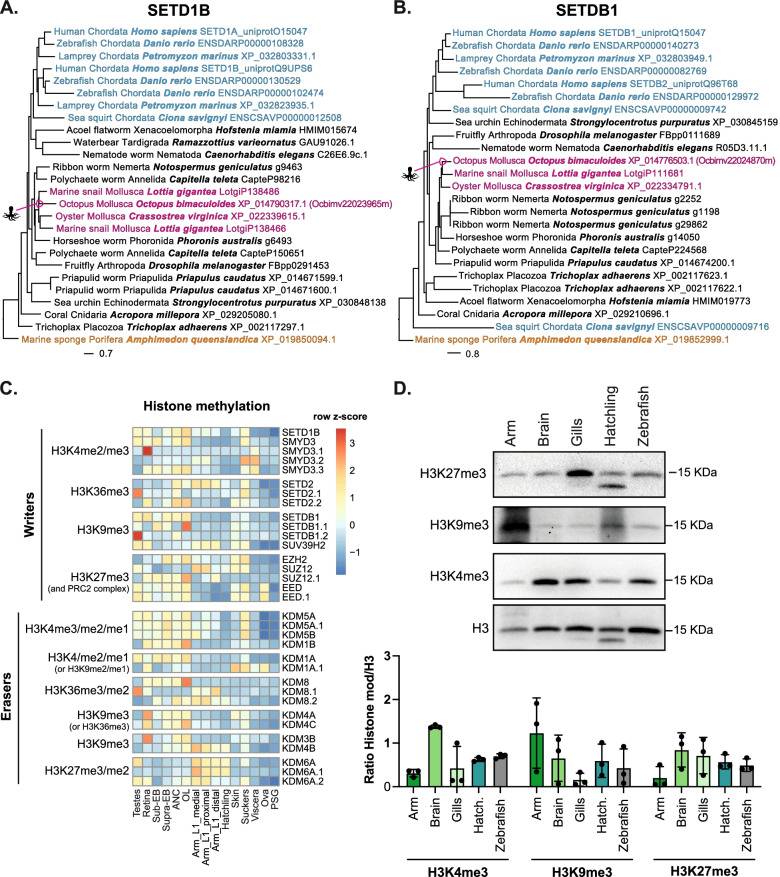
Conserved epigenetic modifiers and histone modifications are present at different levels in octopus tissues. **A** Phylogenetic tree of SETD1B, responsible for H3K4me3 deposition, in a representative subset of 19 metazoan and outgroup species. Colors indicate phyla (blue = Chordata; pink = Mollusca; orange = Porifera), and octopus is indicated with an icon. **B** Phylogenetic tree of SETDB1, responsible for H3K9me3 deposition, in a representative subset of 19 metazoan and outgroup species. Colors indicate phyla (blue = Chordata; pink = Mollusca; orange = Porifera), and octopus is indicated with an icon. **C** Heatmap of the main histone methylation factors, methyltransferase (writers), and demethylases (erasers). **D** Western blot of histone H3 and relative modification (H3K4me3, H3K9me3, H3K27me3) performed on arm (1.5 cm of Right Arm 2), brain (optical lobe), gills, and hatchling of *O. bimaculoides*, in comparison with 5 dpf zebrafish larvae. Quantification of each sample measured by Western blot was normalized to histone H3. Each dot represents one biological replicate, except for hatchling where each dot represents a technical replicate of protein isolated from the same biological sample. Bars represent the mean among replicates, and error bars represent standard deviations

H3K9me3 and H3K27me3 are highly conserved marks of repressed chromatin, while H3K4me3 marks actively transcribed genes, and histone acetylation serves to open chromatin. We investigated the conservation of some well-studied enzymes that write these marks. There was a many-to-one human-octopus ortholog of the H3K4me3 histone methyltransferase (SETD1B in humans; Fig. 5A) and a many-to-one human-octopus ortholog of the H3K9me3 histone methyltransferase (SETDB1 in humans; Fig. 5B); both showing high conservation across metazoans. A many-to-one human-octopus ortholog of the H3K27me3 methyltransferases (human EZH2) is present in octopus and conserved across species (Additional file 18: Fig. S11A). Representative examples from the histone acetylation-modifying enzymes KAT2A and HDAC8 revealed a many-to-one human-octopus ortholog of KAT2A and a one-to-one human-octopus ortholog of HDAC8 in octopus and conserved in other animals (Additional file 18: Fig. S11B-C).

We next examined the expression profile of the genes encoding epigenetic regulators categorized by Trinotate identification (Additional file 3: Table S2) that target histone methylation (Fig. 5C and Additional file 19: S12A) and acetylation (Additional file 19: Fig. S12B). A tissue-specific expression profile was identified for most of these genes, with the testes and neuronal tissue expressing the highest levels of the H3K4 and H3K9 methyltransferases as well as high levels of the H3K4 demethylases (Fig. 5C, Additional file 11: Table S5, Additional file 12: Table S6), while genes that erase H3K27me2/3 were higher in the arms and hatchlings. Interestingly, expression analysis of all histone methyltransferase clearly shows that specific subsets of HMTs characterize a particular tissue (Additional file 19: Fig. S12A). For instance, several of the enzymes that remove methylation from H3K4 (KDM5A/B) are enriched in testes, retina, brain, and suckers, while skin and suckers are enriched for the demethylases that act on H3K9 or H3K36 (i.e., KDM4A/C; Fig. 5C).

### A dynamic pattern of histone modifications in octopus tissues

To determine if these enzymes were functionally active in *O. bimaculoides*, we used Western blotting for H3K27me3, H3K9me3, and H3K4me3 in the arm, brain, and gills of multiple adult octopuses and one 30-day hatchling. This showed a distinct pattern in each tissue: H3K4me3 was detected at highest levels in brain compared to gills and arms, whereas H3K9me3 was higher in arm compared to gills and H3K27me3 was present at comparable level in brain, gills, and hatchling and lower in arms (Fig. 5D, Additional file 20: Fig. S13). This analysis shows that histone modifications are present at different levels in different tissues, suggesting that in octopus, as in many other organisms, the histone code may regulate tissue-specific transcriptomes.

### DNA methylation and histone marks are present in squid and bobtail squid

**Fig. 6:**
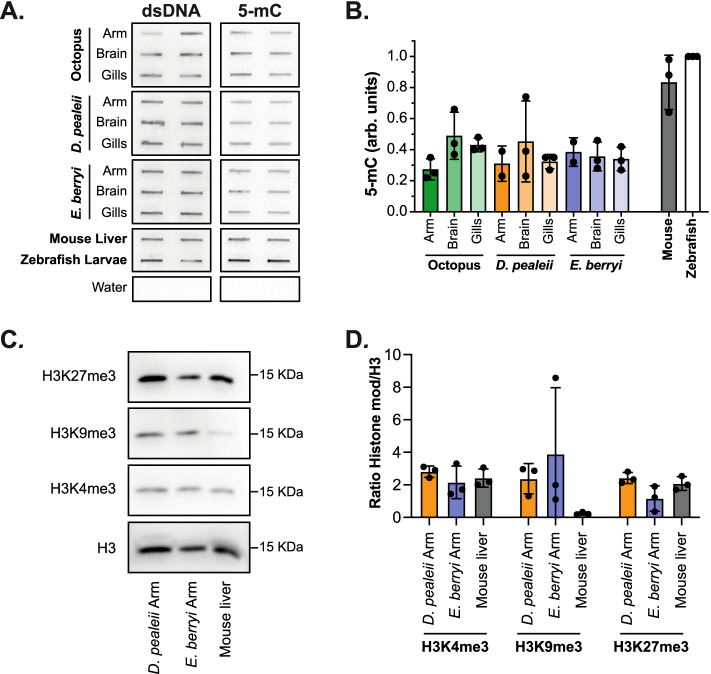
DNA methylation and histone marks are present in squid, and bobtail squid*.***A** Slot blot detecting 5mC on gDNA extracted from arm (2–3 cm of Right Arm 2), brain (optical lobe), gills of one representative animal of *D. pealeii* and *E. berryi*, the analogous tissues of *O. bimaculoides* adults and a 30 dpf hatchling, one representative male mouse adult liver and one representative pool of 5 dpf larvae of zebrafish. Water was used as negative control. **B** Quantification of 5-mC measured by slot blot was normalized to double-stranded DNA (dsDNA), and each sample was normalized to zebrafish larvae as control. Each dot represents one biological replicate, bars represent the mean among replicates, and error bars represent standard deviations. **C** Western blot of histone H3 and modifications on an arm of *D. pealeii* and *E. berryi* and quiescent adult liver from a male mouse. **D** Quantification of each sample measured by Western blot was normalized to histone H3. Each dot represents one biological replicate, bars represent the mean among replicates, and error bars represent standard deviations

To determine if the epigenetic modifications identified in *O. bimaculoides* were conserved in other cephalopods, we analyzed tissues from *D. pealeii* (longfin inshore squid) and *E. berryi* (bobtail squid) for 5mC levels by slot blot and for H3K27me3, H3K9me3, and H3K4me3 by Western blot. 5mC levels in *D. pealeii* and *E. berryi* tissues were similar to those found in *O. bimaculoides* and markedly lower than levels in mouse or zebrafish (Fig. 6A, B, Additional file 21: Fig. S14). Histone marks were detected in arm tissue from both squid and bobtail squid, with H3K27me3 and H3K4me3 at comparable levels to the mouse liver, while H3K9me3 were detected at higher levels in cephalopods compared to mouse (Fig. 6C, D, Additional file 22: Fig. S15). We conclude that the mechanisms that regulate both DNA and histone methylation are conserved in cephalopods.

## Discussion

The role of epigenetics in cell identity, plasticity, development, behavior, and disease is one of the most investigated aspects of biology; however, the role of epigenetics in the distinct transcriptomic profiles in coleoid cephalopods has not been investigated. Moreover, as the 3D genome and genome evolution are dictated by chromatin structure, epigenetics could also contribute to the unique features of cephalopod genomes [26–29, 72, 73]. We described a tissue-specific gene expression pattern in adult octopus and provided an annotation of octopus transcripts that can be integrated with the recently published *O. bimaculoides* genome annotation [27]. Further, this study identified key elements of the cephalopod epigenome, including DNA and histone methylation. We showed that the two main factors required for maintenance DNA methylation in vertebrates, DNMT1 and UHRF1, are evolutionarily conserved, with critical residues necessary for their functions found in human and octopus homologs. The pattern of DNA methylation in octopus resembles the methylome of several other invertebrates, with enrichment on a subset of gene bodies and scant to absent methylation in promoters and transposons. Several histone tail modifications and the genes regulating these modifications are present at very different levels in diverse octopus tissues. Together, this suggests that while DNA methylation is unlikely to be a major factor in regulating transposons potentially contributing to maintain gene expression, histone modifications could be the primary mechanism for regulating gene expression in a tissue-specific fashion in these species.

In most species, the presence of DNMT1 and UHRF1 genes co-occurs with the presence of cytosine methylation [3, 22–24] and is thought to function universally to copy DNA methylation patterns, even in a species of fungi that lack the enzymes that direct de novo methylation [45]. Our finding of a high level of conservation of UHRF1, DNMT1, and detection of 5mC in all tissues evaluated from 3 cephalopod species is consistent with a role for these genes in mediating DNA methylation in these animals. Interestingly, we found that less than 10% of all CpGs are methylated in either developing or adult tissues from *O. bimaculoides.* This is in stark contrast to vertebrates, where over 80% of CpGs are methylated and to popular invertebrate model organisms, which lack DNA methylation entirely. In octopus, methylated CpGs are clustered in the gene bodies of a subset of transcripts and transposons and other repetitive sequences, with the exception of satellites, having very low levels of DNA methylation. Importantly, this pattern is the same in hatchling and adult brain and correlates with genes that have similar levels of expression across many tissues. In contrast, histone methylation levels are different across tissues, suggesting the intriguing idea that genes which are not highly tissue specific are maintained by DNA methylation, whereby those that are dynamically expressed are regulated by a histone code or other epigenetic features.

The approaches used in this study have some limitations which can influence the interpretation of the data. For instance, integrating WGBS and RRBS is limited by the minimal amount of overlap between CpGs common to all datasets, and therefore generalizations about differences in methylome patterning across cell types awaits generation of WGBS datasets from multiple tissues, single cells, and replicate samples of the tissue types analyzed thus far. Single-cell analysis would also be an ideal approach to differentiate between the mix of tissues present in samples from hatchlings. Moreover, as the genomes of other cephalopods have been sequenced recently [26, 27, 73, 74], conclusions about common epigenetic mechanisms across these animals awaits more extensive comparative epigenomic analysis.

Evolution, by convergent or stochastic independent events, has selected for variations in the canonical patterns of methylation [3, 5]. This is illustrated by the high level of DNA methylation across the genome of the sponge *A. queenslandica* [23], the clustering of DNA methylation on repetitive elements in the centipede *S. maritima* [75], and the intermediate pattern of methylation in *C. intestinalis*, where it is high on gene bodies and intermediate on repetitive elements [10, 13, 14]. The pattern of CpG methylation in *O. bimaculoides* appears be more canonical, as it resembles the gene body methylation pattern found in other mollusks [19, 20, 25, 44] and many invertebrates [3, 12, 13, 19, 23]. Importantly, we found bulk 5mC levels to be similar across all tissues sampled from octopus, squid, and bobtail squid. Moreover, the same pattern of DNA methylation is detected in the genomes from different regions of the adult brain and a hatchling, whereby the same genes are categorized as hyper- and hypomethylated in all samples. This implies that methylation patterning is dictated by features intrinsic to the genome, instead of by cell-specific factors.

There are a few caveats that could influence the gene expression and methylome analysis shown here. One is that most tissues only had one replicate for analysis, and this can be particularly confounding when analyzing tissues from different studies, processed with different library preparation protocols and sequenced with different read depth. The exception is for the arm tips which were all processed in our laboratory, where comparison across replicates showed that the gene expression pattern was similar, but not identical. Analysis of additional replicates is a priority for future studies. Features of animal age, sex, and reproductive history could also influence the gene expression pattern. Sexually dimorphic gene expression and epigenomic patterns have been identified in a wide variety of species, including octopus [76], oysters [77], and tunicates [78]. Therefore, the sex of the octopus used in this study could be a contributing factor to the gene expression patterns that we uncovered. Our preliminary transcriptomic analysis of the arms from male and female animals showed few gene expression differences, suggesting that sex may not play a role in influencing gene expression in this tissue.

While gene body methylation is positively correlated with gene expression in organisms as diverse as sponges [23, 24], oysters [19, 25], and mammals [5, 13], it is not clear how this functions. Interestingly, our finding shows that the DNA methylation on transcripts positively correlates with gene expression, with the most unmethylated genes in octopus being silent or expressed at low levels. The striking exceptions to this profile, with some silenced genes being highly methylated and vice versa, argue against DNA methylation as a player in the dynamic regulation of gene expression.

In vertebrates, DNA methylation represses transposons [6]. We [79–81] discovered that zebrafish who have lost DNA methylation have multiple severe phenotypes including embryonic lethality, apoptosis, cell cycle defects, and innate immune activation [82, 83]. These findings are concurrent with studies in sea urchins [84], oysters [85], and an annelid worm [24] where blocking DNA methylation causes developmental defects and prevents regeneration. The phenotypes in zebrafish which lack DNA methylation are in part attributed to activation of TEs [82, 83]. However, while TEs represent about half of the octopus genome [54], there is a stark contrast between high levels of DNA methylation on TEs in vertebrates and virtual absence of TE methylation in cephalopods and most other invertebrates. Despite the obvious threats that transposon expression poses to genome stability, high levels of some transposon transcripts have been detected in octopus brain and other tissues [27, 29, 31], suggesting potential functional relevance of TEs in these animals.

These observations raise important questions, including: what regulates TEs in these species? and, if DNA methylation is not regulating TEs in octopus, what is its function? Answering these questions will require experiments where the DNA methylation machinery can be manipulated in these animals. Recent advances in gene editing in cephalopods [56] provide an exciting new avenue to pursue such studies.

In contrast to the consistency of overall level and pattern of DNA methylation, we show that histone modification levels differ across octopus tissues, as observed in other organisms where specific histone code regulates the tissue-specific transcriptome. The report of H3K4, H3K9, and H3K36 methylation changes during oyster development [86] suggests that these are dynamic and important regulators of mollusk development. An interesting study profiling H3K4me1/me2/me3, H3K36me3, and H3K27Ac in anemone showed that the epigenetic landscape was similar to that found in bilaterians, with conserved regulation for enhancers in this species [87]. Moreover, the finding that the histone pattern in organisms with diverse DNA methylomes recapitulates the vertebrate pattern—such as the planarian *Schmidtea mediterranea*, which lacks DNA methylator complexes and DNA methylation, as well the highly methylated sponge *A. queenslandica* [88, 89]—suggests that many organisms decouple these different marks in patterning the epigenetic landscape. Future studies integrating histone and DNA methylation profiling with transcriptomics in cephalopod tissues can address how these patterns are integrated.

## Conclusions

This work uncovers previously unknown features of the octopus epigenome, which can expand our understanding of epigenetic functionality beyond the few species that are used as a paradigm for knowledge in this field. Studying non-model organisms opens new challenges. For instance, annotation of epigenetic modifiers is based on functions that homologous proteins have in mammals and a thorough functional investigation for each homologous protein should be performed to unequivocally determine their activity in cephalopods. The size and temperate life cycle features of *O. bimaculoides* limits its possibilities for genetic manipulation. Squid, where genetic engineering has been demonstrated [56], and other cephalopod species which are actively being developed as genetic models [28] can serve as alternatives. Identifying the epigenetic patterns of octopus is a first important step in deciphering how these patterns function to regulate the extraordinary features of these animals.

## Methods

### Animal husbandry and sample collection

Cephalopods were maintained in a circulating natural sea water aquaculture facility at the Marine Resources Center at the Marine Biological Laboratory, and all experiments were performed according to the current policy for the use of cephalopods at Marine Biology Laboratories (MBL, https://www.mbl.edu/policies/j110-cephalopod-care-policy). As summarized in Table S1, we used 3 adult male and 1 adult female *O. bimaculoides*, 3 adult male *Euprymna berryi*, and 3 adult *Doryteuthis pealeii* (unknown sex). They were all euthanized in 3% ethanol in natural sea water for 10 min and arm, brain, and gills were dissected, flash frozen in liquid nitrogen, and stored at −80°C. In addition, one 30 days post fertilization hatchling octopus was anesthetized, collected and flash frozen in liquid nitrogen, and stored at −80°C. Samples from *O. bimaculoides* for RNA-seq were obtained from 1 male and 1 female from the first left (L1) arm tip and, from additional 1 adult male from distal, medial and proximal regions of the arm (between 1.5 and 5.5 cm from the arm tip, Additional file 1: Fig. S1A-B). Samples used for RNA-seq, Western blot, or slot blot are indicated in Additional file 2: Table S1.

Zebrafish larvae were generated by incross of wild type adult zebrafish and collected at 5 dpf, euthanized, and flash frozen in liquid nitrogen. Adult fish were raised on a 14:10 h light:dark cycle at 28°C. Mice were maintained in temperature, humidity, and light/dark cycle controlled environment and were fed food and water ad libitum. Mice livers were collected from 8 to 12-week-old male mice (C57Bl/6 background) after euthanasia and flash frozen in liquid nitrogen. All zebrafish (22-0003) and mouse (20-0006A1) protocols were approved by NYU Abu Dhabi for Animal Care and Use Committee (IACUC).

### RNA and DNA extraction

Frozen tissues were ground using a mortar and pestle cooled with liquid nitrogen and placed in dry ice. Fifteen milligrams of tissue powder was used to extract either RNA or DNA. RNA was extracted using Trizol (Invitrogen) following the manufacturer’s instructions with some modifications. Briefly, during precipitation in isopropanol, 10 μg of Glycoblue (Thermo Fisher Scientific) was added and precipitation was performed overnight at −20°C followed by 1 h centrifuge at 12000*g* at 4 °C. RNA was resuspended in water and used in the following procedures. Genomic DNA (gDNA) was extracted by using a DNA extraction buffer (10 mM Tris-HCl pH9, 10 mM EDTA, 200 mM NaCl, 0.5% DSD, 200 μg/ml proteinase K) and overnight incubation at 65°C, followed by RNAase treatment with 2 mg/ml PureLink™ RNase A (Invitrogen) for 2 h at 37°C. Then, 0.25 v/v of 5 M potassium acetate (CH_3_CO_2_K) was added and the sample centrifuged at 12,000×*g* at room temperature to precipitate proteins. 1:1 v/v of isopropanol was added to the supernatant and incubated at −20°C overnight and DNA precipitated by centrifuge at 12,000×*g* at room temperature for 15 min. DNA was resuspended in water and quantified by Qubit dsDNA Broad Range kit.

### Slot blot

Slot blot was performed using 1.5 ng of gDNA that was denatured in 400 mM NaOH/10 mM EDTA and blotted onto nitrocellulose membrane (BioRad) in duplicate for dsDNA and 5mC DNA using a slot blot apparatus (BioRad). Equivalent volume of DNAse/RNAse-free water (Invitrogen) was loaded instead of genomic DNA as negative control. Membranes were incubated 1 h at 80°C, blocked with 5% bovine serum albumin (BSA) in TBST (37 mM NaCl, 20 mM Tris pH 7.5, 0.1% Tween 20), and incubated overnight at 4°C in either anti-dsDNA (Abcam, 1:5000 in 2% BSA in TBST) or anti-5-methyl-cytosine (5mC – Aviva Biosystem clone 33D3, 1:3000 in 2% BSA in TBST). Membranes were washed in TBST and probed with anti-mouse HRP secondary antibody (Promega; 1:2000 in 5% BSA in TBST) for 1 h at room temperature followed by development in ECL (Thermo Fisher Scientific) or Clarity ECL (BioRad). ChemiDoc (BioRad) was used to detect and quantify the chemiluminescent signal. Gel Analyzer (http://www.gelanalyzer.com) was used to perform quantitative densitometric analysis of the signals and ratio between 5mC and dsDNA was plotted for each sample using GraphPad Prism.

### Protein extraction and Western blotting

Frozen tissues were ground using a mortar and pestle cooled with liquid nitrogen and placed in dry ice. Fifteen milligrams of tissue powder was used to extract proteins in lysis buffer (20 mM Tris-HCl, 150 mM NaCl, 1% v/v NP-40, 10% v/v glycerol, 2 mM EDTA). Protein extraction was performed using a probe sonicator (2 s pulse, 2 s pause for 5 min at amplitude 30%), and lysates were cleared by centrifuging at 11,000×*g* for 15 min at 4°C and quantified using Qubit reagent (Invitrogen). For preparation of samples for SDS PAGE, 4× Laemmli buffer (BioRad) was added to protein extracts, incubated at 95 °C for 5 min and 15 μg of proteins was loaded onto 12.5% denaturating gels, electrophoresed, transferred onto PVDF membranes (BioRad), blocked with 5% w/v powdered milk in TBST buffer (20 mM Tris-HCl, 150 mM NaCl, 0.1% v/v Tween 20, pH 8.0) for 1 h at room temperature, and incubated overnight at 4 °C with anti-H3 (SantaCruz, sc-10809, Rabbit polyclonal, 1:5000), anti-H3K4me3 (Abcam, ab8580, Rabbit polyclonal, 1:1000), anti-H3K9me3 (Active Motif, 39161, Rabbit polyclonal, 1:1000), or anti-H3K27me3 (Active Motif, 61017, Mouse monoclonal, 1:1000) diluted in blocking buffer. After washing with TBST and incubation for 1 h with anti-Rabbit IgG HRP Conjugate (Promega, 1:2000) or anti-Mouse IgG HRP Conjugate (Promega, 1:2000) diluted in blocking buffer followed by washing in TBST, membranes were visualized using Pierce™ ECL Western Blotting Substrate (Thermo Fisher Scientific) or Clarity ECL substrate (BioRad) on the BioRad ChemiDoc. Immunoblot bands were quantified by densitometry using GelAnalyzer (http://www.gelanalyzer.com).

### RNA-seq

Total RNA extracted as described above was treated by DNAse I for 30 min at 37 °C followed by RNA purification (RapidOut DNA Removal Kit – Thermo Fisher Scientific). RiboZero was used to remove ribosomal RNA, and the remaining sample was used for library preparation according to the manufacturer’s instructions (Illumina) from 250 ng of RNA. Libraries were sequenced on NextSeq550 (Illumina) to obtain 150 bp paired-end reads. Raw FASTQ sequenced reads were first assessed for quality using FastQC v0.11.5 (http://www.bioinformatics.babraham.ac.uk/projects/fastqc/). The reads were then passed through Trimmomatic v0.36 [90] for quality trimming and adapter sequence removal, with the parameters (ILLUMINACLIP: trimmomatic_adapter.fa:2:30:10 TRAILING:3 LEADING:3 SLIDINGWINDOW:4:15 MINLEN:36). The dataset from hatchling was also processed with Fastp [91] in order to remove poly-G tails and Novaseq/Nextseq-specific artifacts. Following the Fastp quality trimming, the reads were assessed again using FastQC. Quality trimmed reads were used to produce psuedoalignments using Kallisto [92], and the Kallisto quantification was assessed with the --bias flag using the reference *O. bimaculoides* genome (PRJNA270931) and its corresponding annotation. The resulting transcripts per kilobase per million (TPMs) from the pseudo-counts were used for further downstream analysis. Files are available on GEO (accession number: GSE188925) at this link (https://www.ncbi.nlm.nih.gov/geo/query/acc.cgi?acc=GSE188925) [93].

### Trinotate transcriptome annotation

The quality trimmed reads were aligned to the *O. bimaculoides* genome (PRJNA270931) using HISAT2 [94] with the default parameters and additionally by providing the –dta flag. The resulting SAM alignments were then converted to BAM format and coordinate sorted using SAMtools v1.3.1 [95]. The sorted alignments were processed through Stringtie v1.3.0 [96] for transcriptome quantification to produce a GTF file per sample. The GTFs were then combined using STRINGTIE merge to produce one merged GTF representing the transcriptome for the genome. Finally, Qualimap [97] v2.2.2 was used to generate RNA-seq specific QC metrics per sample. Following the transcriptome quantification steps above, the Trinotate [98] pipeline was used to annotate the transcriptome, in addition to the existing reference annotation. The Trinotate steps as detailed in the software’s user manual were followed. Briefly, after transcriptome preparation, BlastP longest ORFs using the longest_orfs protein sequences against the Uniprot database and Pfam domain search using longest ORFs against the Pfam database were integrated into coding region selection using TransDecoder.Predict. In addition, the transcriptome FASTA file was BlastX against the Uniprot database and domain scanned using HMMscan to generate gene to transcript mappings using transIDmapper.pl with the output exported to an SQLite database.

### Reduced representation bisulfite sequencing (RRBS)

RRBS was performed on gDNA extracted from the same 30 dpf hatchling of *O. bimaculoides* used in RNA-seq. Briefly, 1000 ng of genomic DNA was digested with 200 U of MspI (New England Biolabs) for 24 h at 37°C. Digested DNA was used for preparing the library as previously described [99], with the exception that adaptors used for multiplexing were purchased separately (Next Multiplex Methylated Adaptors - New England Biolabs). Libraries were size-selected by dual-step purification with Ampure XP Magnetic Beads (Beckman Coulter, Agencourt) to specifically select a range of fragments from 175 to 670 bp, as previously described [83]. Bisulfite conversion was performed with Lightning Methylation Kit (ZYMO Research) by following the manufacturer’s instructions. Libraries were amplified using KAPA HiFi HotStart Uracil+ Taq polymerase (Roche) and purified with Ampure XP Magnetic Beads (Beckman Coulter, Agencourt) before sequencing. Libraries were sequenced using the Illumina Nextseq550 sequencer. Quality control was undertaken using FASTQC (http://www.bioinformatics.babraham.ac.uk/projects/fastqc). Reads were quality trimmed using Trimmomatic [90] to remove low-quality reads and adapters. Reads passing quality control were aligned to the reference genome (assembly PRJNA270931, available here: https://groups.oist.jp/molgenu/octopus-genome) using default parameters in Bismark [100], which adopts Bowtie2 as the aligner [101] and calls cytosine methylation at the same time. Fastq files are available on BioSample (accession number: SAMN23139394) at this link (https://www.ebi.ac.uk/biosamples/samples/SAMN23139394) [102]. RRBS metrics describing lengths of reads, numbers of sequenced and mapped reads, and identified cytosines were extracted using ‘bismark2report’ function in Bismark (Additional file 23: Table S8A), while conversion rates (99.20%) and coverage stats were extracted using ‘processBismarkAln’ and ‘getCoverageStats’ function in methylKit (Additional file 23: Table S8B) [103].

### Bioinformatic analysis

RNA-seq data was analyzed as described above and visualized in RStudio (R version 4.0). Heatmaps for transcriptomic profiling were performed by using R package ‘pheatmap’. Clusters were calculated on the hierarchical clustering of dendrogram (Euclidean distance) based on the normalized expression profile (row *z*-score across different tissues). The number of clusters was determined based on the optimal performance to discriminate the different tissues used for the analysis. For Gene Ontology (GO), GO terms were downloaded from Ensemble Metazoa (BioMart database). GO enrichment analysis was conducted using the GO hypergeometric over-representation test in the ‘ClusterProfiler’ package in R. An adjusted *p*-value < 0.05 was treated as significant for all analyses. Unique stable transcript IDs were annotated with GO terms from BioMart database and divided by Molecular Function, Biological Process, and Cellular Component terms. Putative members of epigenetic machinery represented in the heatmaps were identified using Trinotate as described above and reported with corresponding human gene symbols. For RRBS analysis on hatchling samples, CpG methylation levels were extracted from Bismarck aligned file with the R package ‘methylKit’ [103]. CpGs covered at least 10 times (and with minimum phred quality score = 20) were included in the analysis. Whole genome bisulfite sequencing (WGBS) data from the Supra and Sub Esophageal brain regions were obtained from public available GEO datasets (GSE141609) [104]. Specifically, Supra (GSM4209498) and Sub (GSM4209499) esophageal brain Bisulfite-seq files (CGmap files) were downloaded and used as source of CpGs methylation analysis. To compare WGBS data with RRBS, CGmap files were processed as follows: CGmap files were filtered for methylation on CpG context only (based on column 4 representing the context (CG/CHG/CHH)); strand direction information was obtained converting C into + and G into − (based on column 2 representing the nucleotide on Watson (+) strand); percentage of methylation was obtained multiplying by 100 the methylation level (from 0 to 1; in 0 to 100 values) (based on column 6 methylation level). CpGs with methylation levels below 20% were treated as unmethylated and above 80% were considered as methylated. Genomic element annotation and metaplots of CpGs were performed with R package ‘genomation’. Repetitive elements (RE) were identified using the Repeat Masker annotation on the reference genome (assembly PRJNA270931, available here: https://groups.oist.jp/molgenu/octopus-genome), and manually curated to group the classes of transposons (DNA, LTR, LINE, SINE) and non-transposons (Satellite, Simple Repeats, Other RE). Since sequences represented (transcripts or RE) are of unequal length, metaplots were divided into 30 or 15 equal bins (based on the average length of the sequences analyzed). Lines in metaplots represent the winsorized mean values (1–99 percentile) for each bin, and blue shades represent dispersion of 95% confidence interval for the mean. Heatmaps of DNA methylation pattern were performed with R package ‘EnrichedHeatmap’ on the full-length transcripts and 2000 bp upstream and downstream. DNA methylation average of gene bodies for each transcript was divided into 4 clusters based on k-means from R package ‘stats’. Number of clusters was optimized at 4 based on the ability to discriminate between the distinct patterns of DNA methylation. Center of k-means from Patterns 1 to 4 are respectively 71.06, 49.11, 28.59, and 1.31. Subsequent analyses on methylation pattern were performed using R package ‘ggplot’ for violin plots, ‘pheatmap’ for expression profiling, and ‘ClusterProfiler’ for GO enrichment analysis. All code used for this study is available on Github (https://github.com/SadlerEdepli-NYUAD/Macchi-et-al-2022-Cephalopod-DNA-Methylation) [105].

### Phylogenomic analysis

An phylogenomic pipeline was built, based in part on a prototype of the GIGANTIC pipeline [64]. Human sequences for genes encoding DNA methylation and histone modification factors were collected from Uniprot (October 5, 2021) to generate reference gene sets (RGS) per family (Additional file 24: Table S9). Project databases, Metazoa50 and Metazoa19 (a subsample of Metazoa50), representing 50 and 19 animal and unicellular outgroup genomes, respectively, were generated per genome (Additional file 7: Table S4). Genomes were sourced from Ensembl, NCBI RefSeq, or other public databases (Additional file 7: Table S4), and species and data set selection was based on representation of major clades and phyla and BUSCO-based evaluations of genome quality (BUSCO; Metazoa10) [106]. Genome gene models were filtered to retain single longest protein isoform per coding gene locus. Fasta file headers were standardized per genome based on NCBI Taxonomy (January 2021) details per species and on common names as provided in Wikipedia (October 2021) (Additional file 7: Table S4). Blastp databases were generated per genome using NCBI Blast+ (version 2.6.0) Makeblastdb. Metazoa50 genomes and RGS sequences were domain annotated using Interproscan Pfam (version 5.48-83.0) [107].

Human reference gene sets were blasted against Metazoa19 and Metazoa50 Blast databases per gene family or superfamily using NCBI Blast+ Blastp (e-5 cutoff). All hits were then blasted against the human genome and top hits to any human RGS sequence retained to form the candidate gene set (CGS), which were combined with RGS sequences to form a final gene set. Sequences were aligned in MAFFT (linsi command; version 7.487) [108], alignments were trimmed in ClipKIT (super gappy; version 1.1.3) [109], and maximum likelihood trees were built in IQTREE2 (with ModelFinder; version 2.1.2) [110]. Alignments and trees were visually assessed in Geneious (version 2021.1.1) and in FigTree (version 1.4.4 and iTOL (version 6) [111], respectively. Sequences residing on UHRF1, DNMT1, SETD1B, SETDB1, EZH2, KAT2A, and HDAC8 branches within a larger superfamily tree were collected and then aligned and trimmed, and a tree was built for just the family to improve branching structure and support relative to the superfamily tree. Octopus sequences came from the NCBI RefSeq genome and were mapped to the Ensembl genome based on top Blastp hit to match sequence identifier to those used in transcriptome analyses. Trees were color annotated in FigTree and rooted on sponge or a unicellular outgroup or else left unrooted. All dataset and code used for this study are available on Dryad at this link (10.5061/dryad.d51c5b069) [112].

### Protein alignment

Protein sequences were downloaded from the UniProt database (https://www.uniprot.org) as FASTA formatted files and alignments were performed using ClustalOmega with multiple sequence alignment program [113]. Output alignment files generated in a ClustalW format with character counts were reformatted and colored based on amino acid residue identity using MView (https://www.ebi.ac.uk/Tools/msa/mview/). Protein sequences of interest were processed with phmmer [114] and queried against HMM target databases using profile hidden Markov models. Sequence matches were calculated by multiple factors, grouped by Pfam domains, and homology sequence probability were represented by Bit score.

### Swiss-Expasy 3D Model

To build protein homology models, we used the Automated Mode (https://swissmodel.expasy.org/docs/help) of the SWISS-MODEL server homology modeling pipeline [68]. In brief, homology modeling proceeds through four main steps: (i) alignment of target sequence and identification of structural templates by BLAST and HHblits; (ii) alignment and sorting of target–template structures based on Global Model Quality Estimation (GMQE) and Quaternary Structure Quality Estimation (QSQE); (iii) model-building relying on ProMod3 [115] and OpenStructure comparative modelling engine [116]; and (iv) model quality evaluation using GMQE estimation score, and another composite estimator QMEAN [117]. SWISS-MODEL Structure comparison tool was used to perform super-positioning of newly computed 3D models of *O. bimaculoides* proteins and published structures of mouse DNMT1 (PDB ID 4da4.1) and UHRF1 (PDB ID 2zke.1).

### Statistical analysis

All experiments were carried out on at least 3 biological replicates, except where noted. For slot blot analysis, technical replicates were also included. The number of replicates for each experiment is indicated in each figure. Methods to evaluate statistical significance include unpaired parametric one-way ANOVA test adjusted with Tukey’s multiple comparisons test or unpaired non-parametric Kruskal-Wallis test adjusted with Dunn’s multiple comparisons test. Tests used are indicated in each graph. All plots were generated in GraphPad Prism 9 or RStudio (R version 4.0). Statistical analysis was performed in GraphPad Prism 9.

## Supplementary Information

**Additional file 1: Figure S1.** Representative images of *O. bimaculoides.* A. Picture of anesthetized adult male. B. Representative image of *O. bimaculoides* arm for collection of distal, medial and proximal samples. C. A clutch of 30 dpf hatchlings and D. the hatchling used for DNA, RNA and protein extraction.

**Additional file 2: Table S1.** List of all the samples with the origin and the usage in this study.

**Additional file 3: Table S2.** Trinotate table containing the putative gene names of the transcriptome of *O. bimaculoides*.

**Additional file 4: Table S3.** Table of Ensembl transcript IDs contained in each tissue specific cluster.

**Additional file 5: Figure S2.** Comparison of RNA-seq on octopus arms between females and males. A. Venn Diagram of the 1405 genes from a unified set of the top 1000 expressed transcripts (TMP) between the tips of L1 arm of 1 male and 1 female, and a more distal region of the arm of another male. B. Heatmap of Log2(TMP+1) of the transcripts identified on A.

**Additional file 6: Figure S3.** Distinct biological processes and cellular components of differentially expressed genes octopus tissues. GO analysis of A. Biological Process and B. Cellular Component of each gene cluster identified in Fig. 1.

**Additional file 7: Table S4.** Genome sources and details of organisms utilized in phylogenomic pipeline.

**Additional file 8: Figure S4.** Extended phylogenetic analysis shows high conservation of DNMT1 and UHRF1. A. Phylogenetic tree of DNMT1 in a representative subset of 50 metazoan and outgroup species. Colors indicate phyla (blue = Chordata; pink = Mollusca; orange = Porifera; purple = Ctenophora) and octopus is indicated with an icon. B. Phylogenetic tree of UHRF1 in a representative subset of 50 metazoan and outgroup species. Colors indicate phyla (blue = Chordata; pink = Mollusca; orange = Porifera; purple = Ctenophora) and octopus is indicated with an icon.

**Additional file 9: Figure S5.***O. bimaculoides* genome encodes conserved features of DNMT1 and UHRF1 but not UHRF2. A. Similarity for each domain of *O. bimaculoides* DNMT1 determined by HMMER. Bit score indicates homology score. B. Table shows percentage of sequence identity calculated by ClustalOmega for the *O. bimaculoides* DNMT1 protein compared to human, mouse, and zebrafish in a multiple sequence alignment. C. Table shows similarity for each domain (with a PFAM ID) of *O. bimaculoides* UHRF1 when compared to the proteome database using HMMER. Bit score indicates homology score. D. Table shows percentage of sequence identity calculated by ClustalOmega for the *O. bimaculoides* UHRF1 protein compared to human, mouse, and zebrafish in a multiple sequence alignment. E. Similarity for each domain of the *O. bimaculoides* YDG_OCTBM protein determined using HMMER. Bit score indicates homology score. F. Percent identity calculated by ClustalOmega for the *O. bimaculoides* YDG_OCTBM protein compared to human, mouse, and zebrafish in a multiple sequence alignment. G. Alignment of the SRA domain in *O. bimaculoides* YDG_OCTBM to human, mouse, and zebrafish shows that all the major residues needed for the correct functionality of UHRF1 are not conserved in YDG_OCTBM of *O. bimaculoides*. Alignment to UHRF2 shows no conservation of critical residues between YDG_OCTBM in *O. bimaculoides* and UHRF2 in human, mouse. Residues functionality is based on mouse orthologs.

**Additional file 10: Figure S6.** Structural modelling of DNMT1 and UHRF1. A. 3D structure of the BAH1, BAH2 and CTD domains of DNMT1 in *M. musculus*. B. 3D model of BAH1, BAH2 and CTD domains of DNMT1_OCTBM in *O. bimaculoides*. C. 3D structure of SRA domain of UHRF1 in *M. musculus*. D. 3D model of SRA domain of UHRF1_OCTBM in *O. bimaculoides*.

**Additional file 11: Table S5.** Table of Ensembl transcript IDs contained in each heatmap of epigenetic factors. The mapping across different identifiers is also reported for each transcript.

**Additional file 12: Table S6.** Table of TMP count used to generate z-scores in each heatmap of epigenetic factors.

**Additional file 13: Figure S7.** Original uncropped images of Slot blot in Fig. 3A. A. Blots containing biological replicate 1 and used for quantification. B. Blots containing replicate 2 and run with water used to prepare all samples and solutions.

**Additional file 14: Figure S8.** Pattern of DNA methylation identified by WGBS and RRBS in octopus tissues. A. Table describing the number and relative percentage of CpGs covered in the *O. bimaculoides* genome by each technique and sample analyzed. CpGs were classified based on the percentage of methylation as methylated (> 80%) or not methylated (< 20%). B. Scaled violin plot of CpGs identified by WGBS and RRBS in Supra E and Sub E brain and in one whole-body 30 dpf hatchling. Box-and-whisker inside violin plots have a center line at the median, lower and upper hinges correspond to first and third quartiles, and whiskers extend from hinges to largest or smallest values no further than 1.5 × IQR (inter-quartile range), while data beyond the end of the whiskers are outlying points that are plotted individually. Numbers on the lines indicate the percent of CpGs that are detected as >80% methylated. C. Scatter plot of DNA methylation levels of common CpGs across Supra Esophageal (Supra E) and Hatchlings. Dot color represents DNA methylation levels in Sub Esophageal (Sub E) and size of the dots indicates scaled proportion of CpGs represented by each dot. D. Scatter plot of DNA methylation levels of common CpGs across Sub Esophageal (Sub E) and Hatchlings. Dot color represents DNA methylation levels in Supra Esophageal (Supra E) and size of the dots indicates scaled proportion of CpGs represented by each dot.

**Additional file 15: Figure S9.** Pattern of DNA methylation identified by WGBS and RRBS in repetitive elements. A. Box plot describing the percentage of methylation of CpGs contained in repetitive elements (RE) divided by class in the 30 dpf hatchling. B. Box plot depicting the percentage of methylation of CpGs contained in repetitive elements (RE) divided by class in the 30 dpf hatchling and down-sampled based on the number of CpGs in satellite repeats. Box-and-whisker plots have a center line at the median, lower and upper hinges correspond to first and third quartiles, and whiskers extend from hinges to largest or smallest values no further than 1.5 × IQR (inter-quartile range), while data beyond the end of the whiskers are outlying points that are plotted individually. C. Metaplot displays CpG methylation levels of intergenic TEs, intergenic non-transposable repetitive elements and in non-repetitive intergenic regions in Supra E and Sub E brain and hatchling. D. CpG density of the same regions defined in panel C. Each region is divided in 15 bins.

**Additional file 16: Table S7.** Table of Ensembl transcript IDs contained in each Pattern.

**Additional file 17: Figure S10.** Relationship between DNA methylation pattern gene expression, and gene length in octopus. A. Heatmap of DNA methylation of all full-length transcripts and 2000 bp upstream and downstream as detected in the Sub E brain WGBS data. Clustering and rank order is dictated by Supra E brain samples shown in Fig. 4A. B. Heatmap of DNA methylation in hatchling full-length transcripts and 2000 bp upstream and downstream. Clustering and rank order is dictated by Supra E brain samples shown in Fig. 4A. C. Overall DNA methylation of transcripts (TSS to TTS of each transcript) in Sub E brain and hatchling is regressed against transcripts expression (log2(TPM+1)). Transcripts were grouped by percentile of expression values and each dot represents the average value of DNA methylation for each percentile. D. Violin plot displays the distribution of transcript length (as log10(width)) in each methylation pattern. Box-and-whisker inside violin plots have a center line at the median, lower and upper hinges correspond to first and third quartiles, and whiskers extend from hinges to largest or smallest values no further than 1.5 × IQR (inter-quartile range), while data beyond the end of the whiskers are outlying points that are plotted individually. *p*-values were calculated by unpaired non-parametric Kruskal-Wallis test adjusted with Dunn’s multiple comparisons test. **** indicates *p*-value adjusted < 0.0001.

**Additional file 18: Figure S11.** Supplemental phylogenetic trees. A. Phylogenetic tree of EZH2, responsible of H3K27me3 deposition, in a representative subset of 19 metazoan and outgroup species. B. Phylogenetic tree of KAT2A, mainly responsible of H3K9ac deposition, in a representative subset of 19 metazoan and outgroup species. C. Phylogenetic tree of HADC8, mainly responsible of H3K9ac removal, in a representative subset of 19 metazoan and outgroup species. Colors indicate phyla (blue: Chordata, pink: Mollusca, orange: Porifera; ocra: Nematoda), and octopus are indicated with an icon.

**Additional file 19: Figure S12.** Histone methyltransferases, acetyltransferases and de-acetylases have a tissue specific expression pattern in octopus. A. Heatmap of the extended panel of histone methyltransferases. B. Heatmap of the main histone acetylation factors, acetyltransferase (writers) and de- acetylases (erasers).

**Additional file 20: Figure S13.** Original uncropped images of western blot in Fig. 5D.

**Additional file 21: Figure S14.** Original uncropped images of Slot blot in Fig. 6A. A. Blots containing biological replicate 2 and used for quantification. B. Blots containing replicate 3 and run with water used to prepare all samples and solutions.

**Additional file 22: Figure S15.** Original uncropped images of western blot in Fig. 6C.

**Additional file 23: Table S8.** Statistical metrics of RRBS. A. Statistic values of alignment performed in Bismark. B. Read coverage statistics per base analyzed by methylKit.

**Additional file 24: Table S9.** Reference Gene Set (RGS) sources and details of proteins utilized in phylogenomic pipeline.

## Data Availability

The datasets generated during the current study are available in the GEO repository (accession number: GSE188925) at this link (https://www.ncbi.nlm.nih.gov/geo/query/acc.cgi?acc=GSE188925) for RNA-seq [93], and on BioSample database (accession number: SAMN23139394) at this link (https://www.ebi.ac.uk/biosamples/samples/SAMN23139394) for RRBS [102]. All the codes to analyze genomic data used in this paper are available in Github at this link (https://github.com/SadlerEdepli-NYUAD/Macchi-et-al-2022-Cephalopod-DNA-Methylation) [105], and the code and dataset used for evolutionary analysis are available in Dryad at this link (10.5061/dryad.d51c5b069) [112].

## Abbreviations

WGBS: Whole genome bisulfite sequencing
RRBS: Reduced representation bisulfite sequencing
5mC: 5-Methyl-cytosine
dpf: Days post fertilization
UHRF1: Ubiquitin Like With PHD And Ring Finger Domains 1
DNMT1: DNA methyltransferase 1
H3K4me3: Tri-methylation at the 4th lysine residue of the histone H3
H3K9me3: Tri-methylation at the 9th lysine residue of the histone H3
H3K27me3: Tri-methylation at the 27th lysine residue of the histone H3
