# Supplementary figures and images for: Epigenetic machinery is functionally conserved in cephalopods
Source: BMC Biol. 2022 Sep 14;20:202. doi: 10.1186/s12915-022-01404-1 (PMC9476566; doi:10.1186/s12915-022-01404-1)

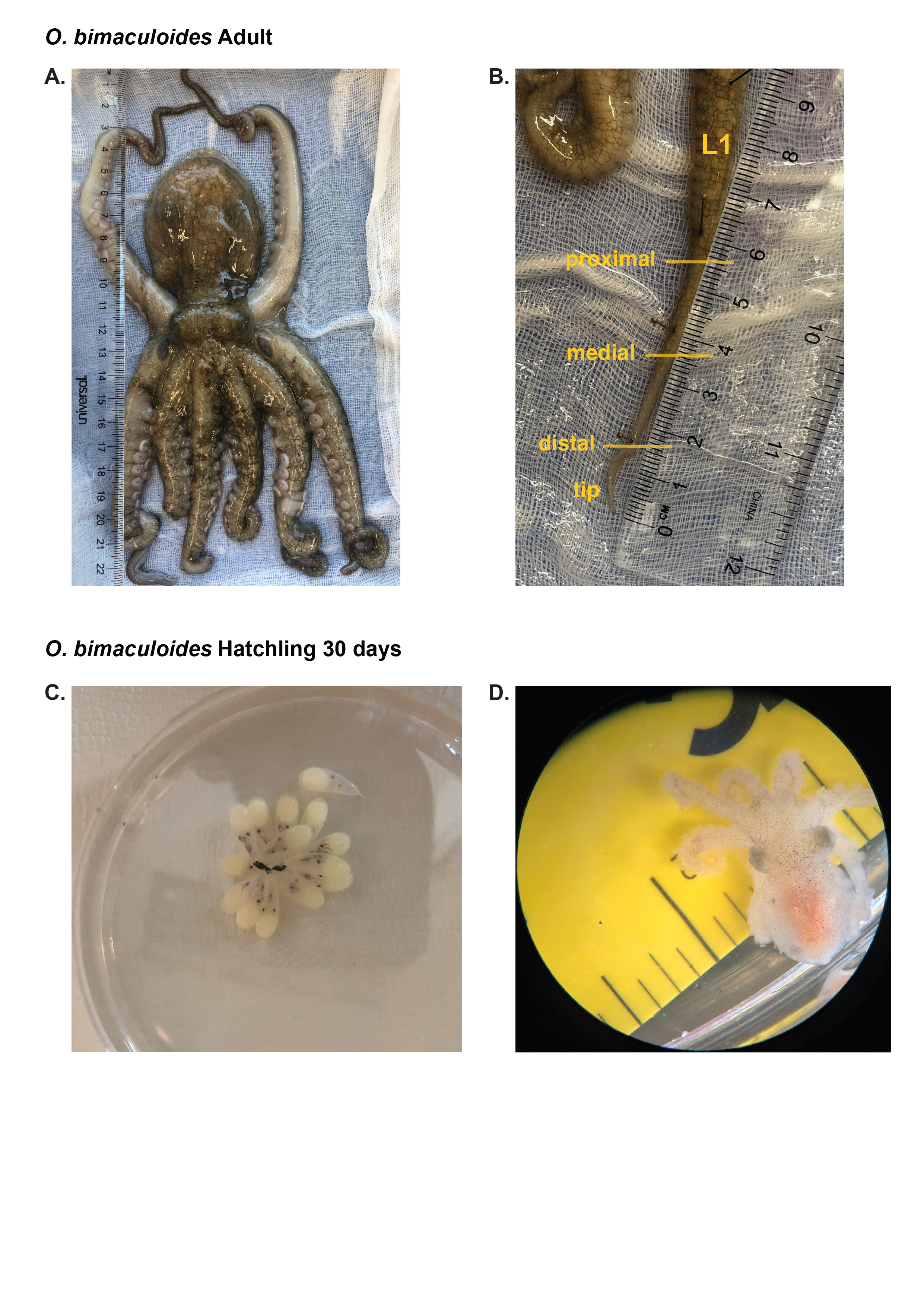

Supplement: Supplementary file 1 — Additional file 1: Figure S1. Representative images of O. bimaculoides. A. Picture of anesthetized adult male. B. Representative image of O. bimaculoides arm for collection of distal, medial and proximal samples. C. A clutch of 30 dpf hatchlings and D. the hatchling used for DNA, RNA and protein extraction. [file 12915_2022_1404_MOESM1_ESM.png]

**A.** **Top 1000 expressed transcripts**

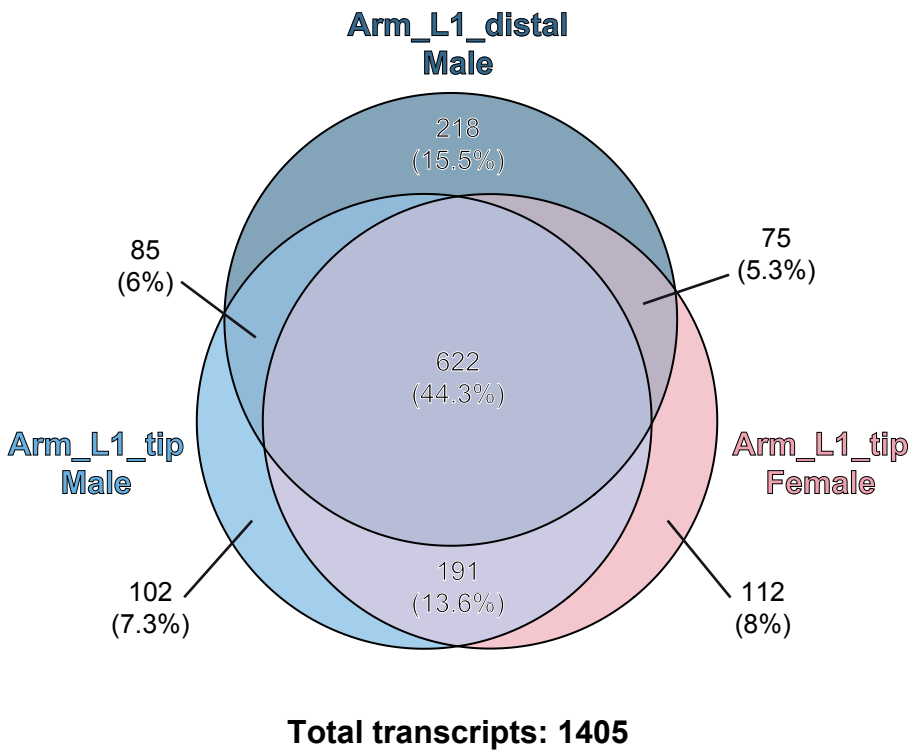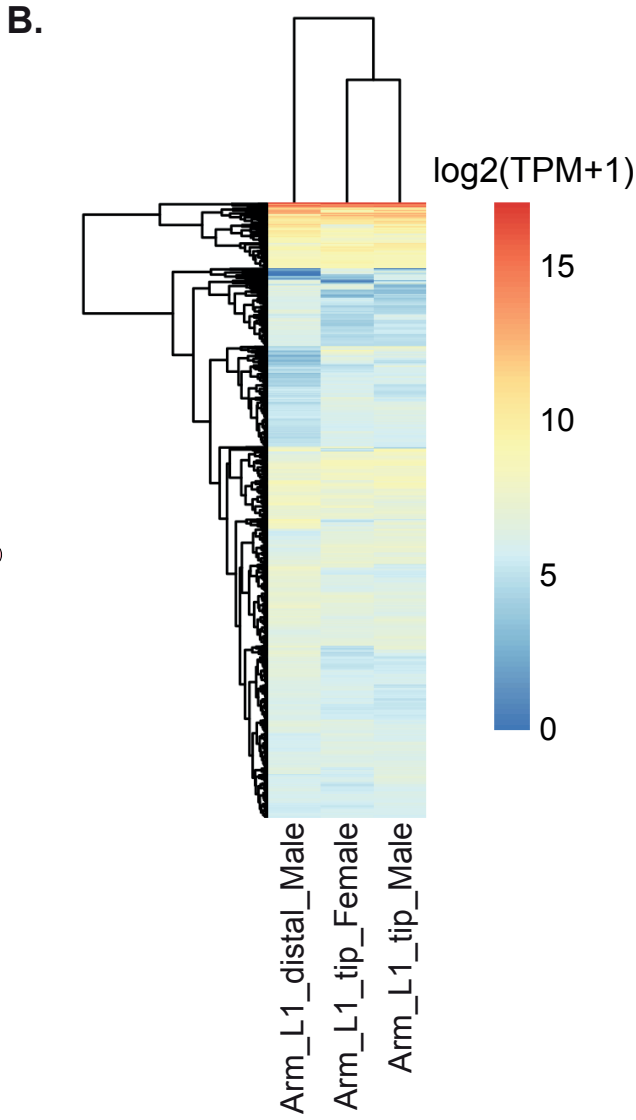

Supplement: Supplementary file 5 — Additional file 5: Figure S2. Comparison of RNA-seq on octopus arms between females and males. A. Venn Diagram of the 1405 genes from a unified set of the top 1000 expressed transcripts (TMP) between the tips of L1 arm of 1 male and 1 female, and a more distal region of the arm of another male. B. Heatmap of Log2(TMP+1) of the transcripts identified on A. [file 12915_2022_1404_MOESM5_ESM.pdf]

## A. GO Biological Process

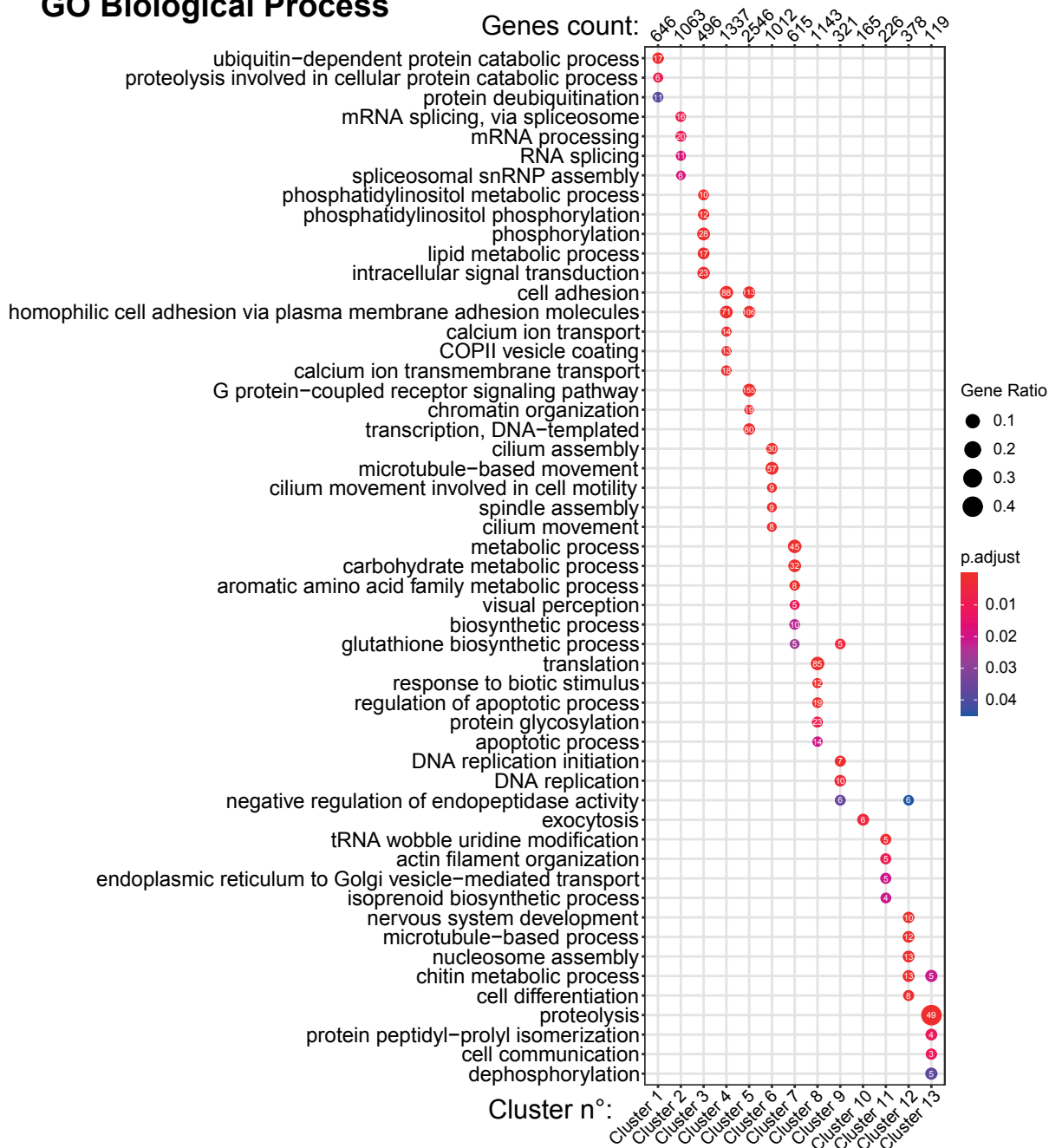

## B. GO Cellular Component

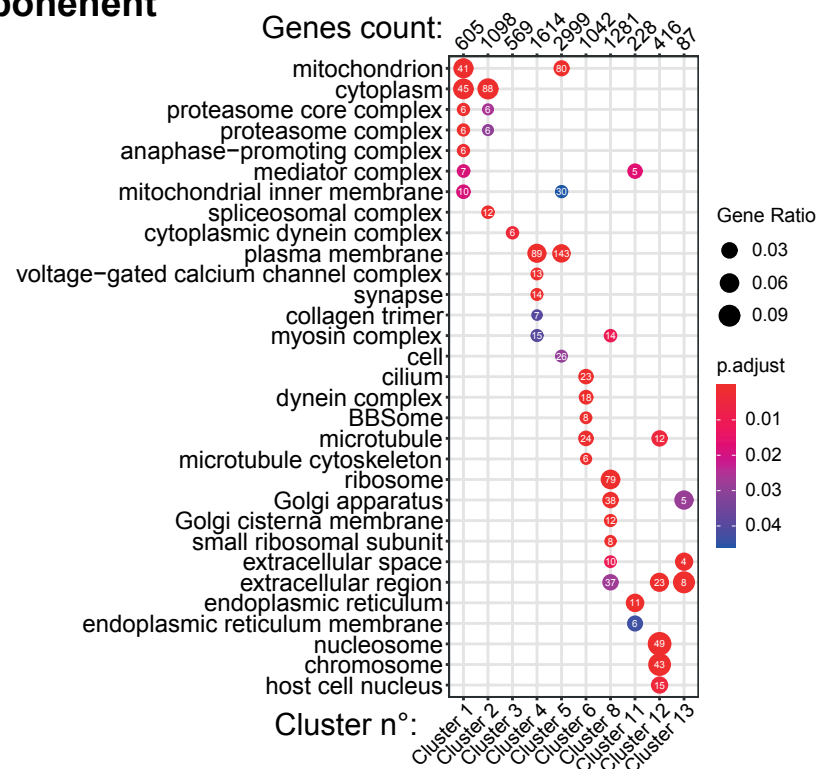

Supplement: Supplementary file 6 — Additional file 6: Figure S3. Distinct biological processes and cellular components of differentially expressed genes octopus tissues. GO analysis of A. Biological Process and B. Cellular Component of each gene cluster identified in Fig. 1. [file 12915_2022_1404_MOESM6_ESM.pdf]

A. Metazoa 50: DNMT1

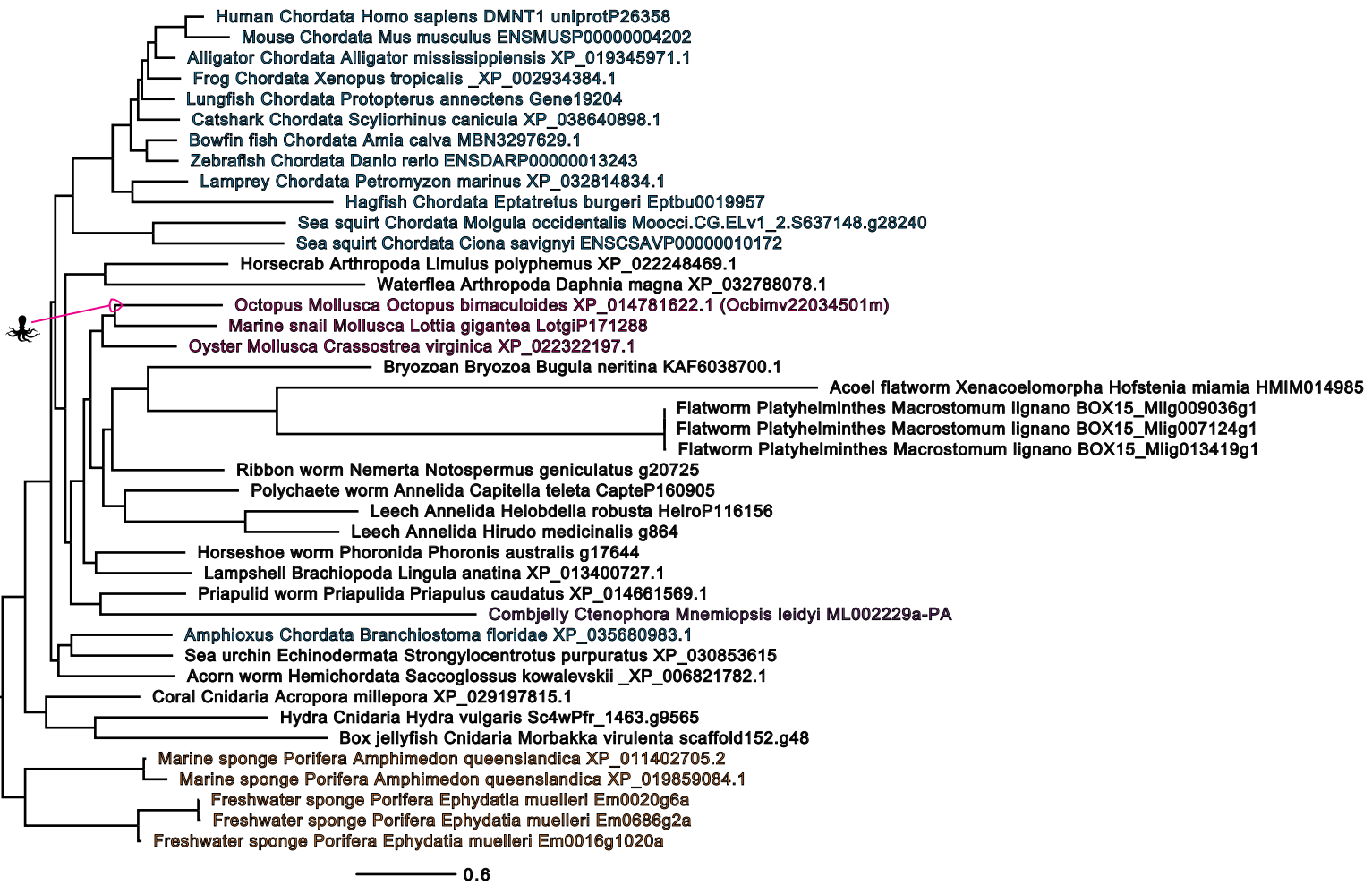

B. Metazoa 50: UHRF1

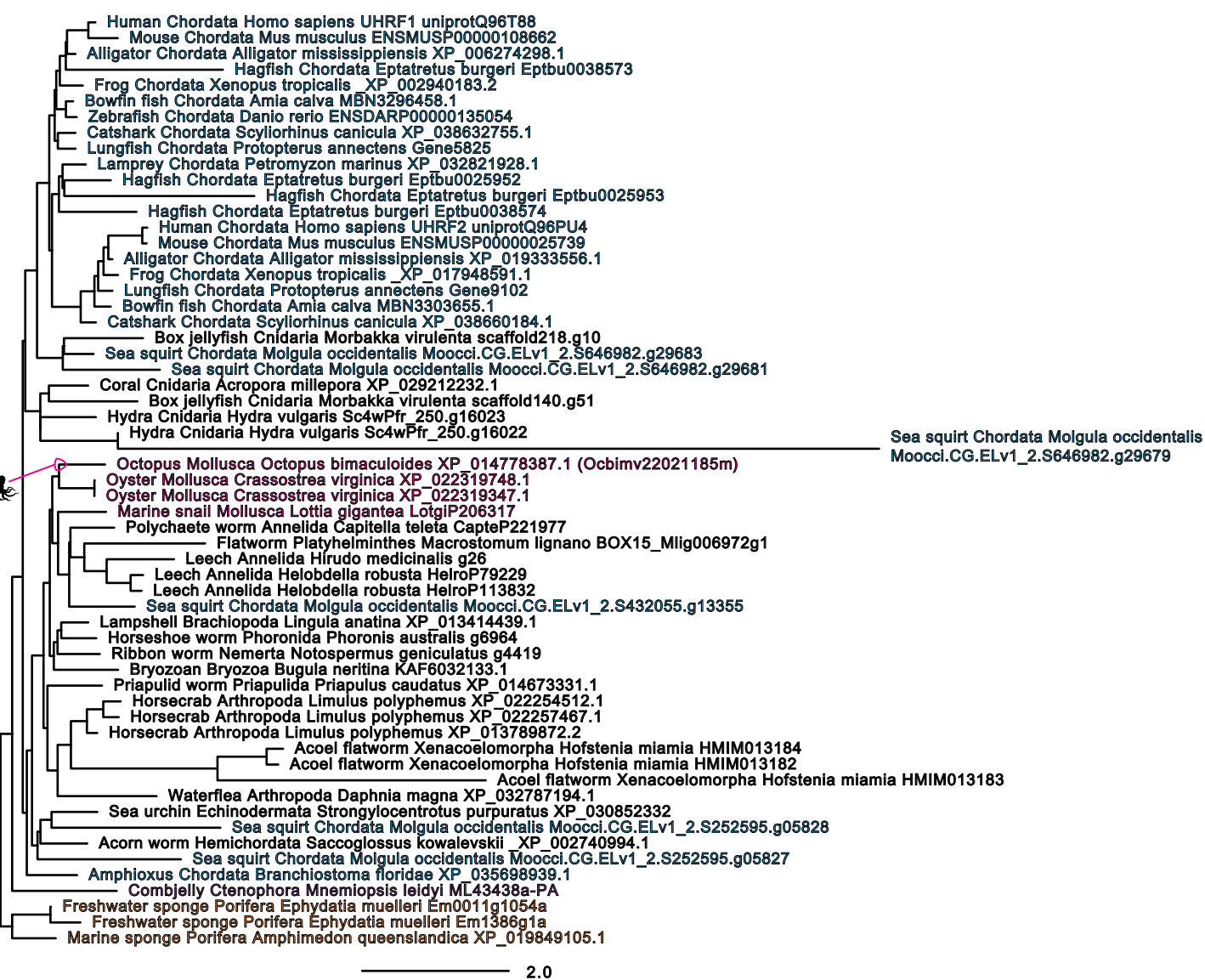

Supplement: Supplementary file 8 — Additional file 8: Figure S4. Extended phylogenetic analysis shows high conservation of DNMT1 and UHRF1. A. Phylogenetic tree of DNMT1 in a representative subset of 50 metazoan and outgroup species. Colors indicate phyla (blue = Chordata; pink = Mollusca; orange = Porifera; purple = Ctenophora) and octopus is indicated with an icon. B. Phylogenetic tree of UHRF1 in a representative subset of 50 metazoan and outgroup species. Colors indicate phyla (blue = Chordata; pink = Mollusca; orange = Porifera; purple = Ctenophora) and octopus is indicated with an icon. [file 12915_2022_1404_MOESM8_ESM.pdf]

A.

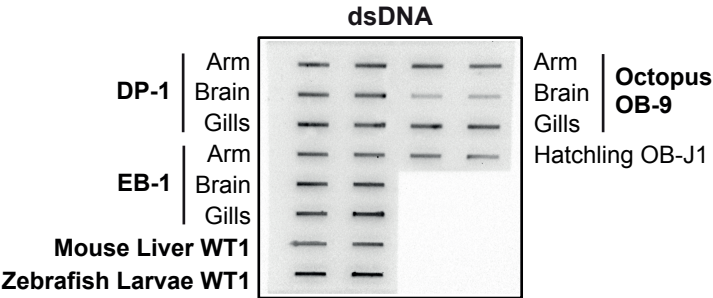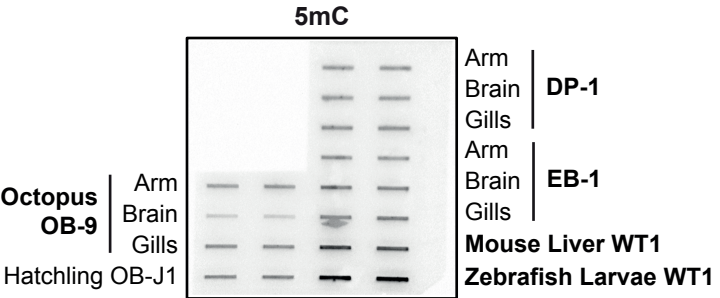

B.

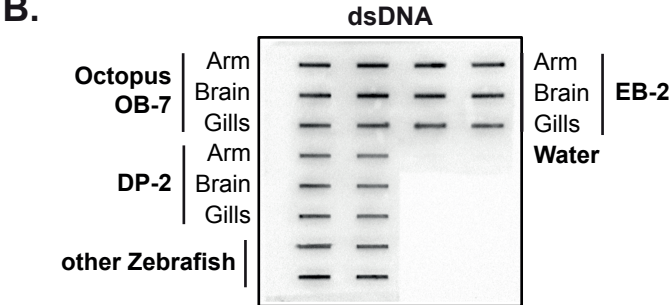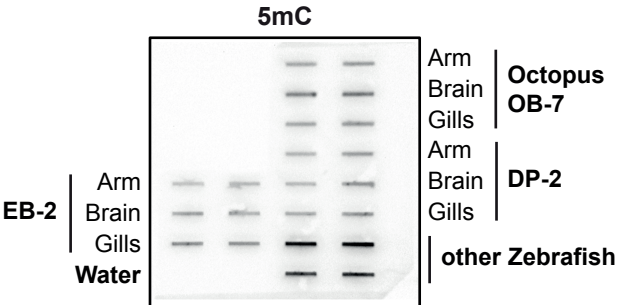

Supplement: Supplementary file 13 — Additional file 13: Figure S7. Original uncropped images of Slot blot in Fig. 3A. A. Blots containing biological replicate 1 and used for quantification. B. Blots containing replicate 2 and run with water used to prepare all samples and solutions. [file 12915_2022_1404_MOESM13_ESM.pdf]

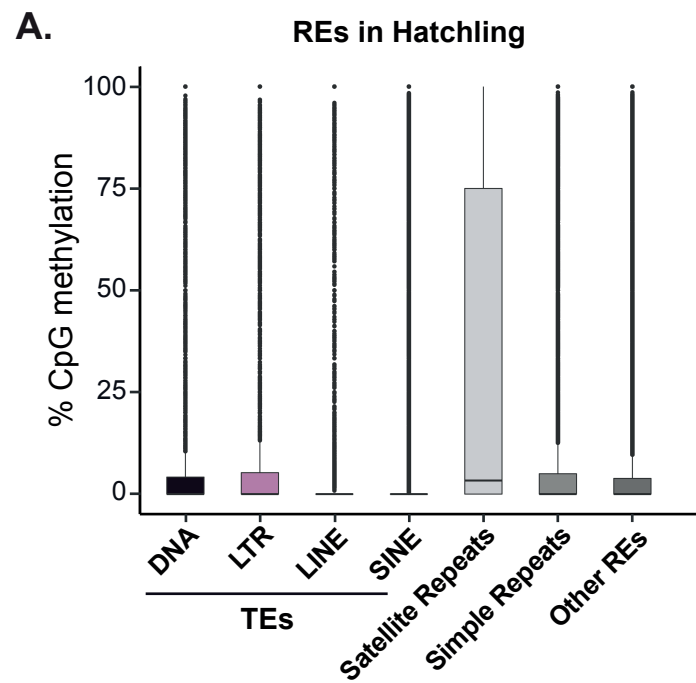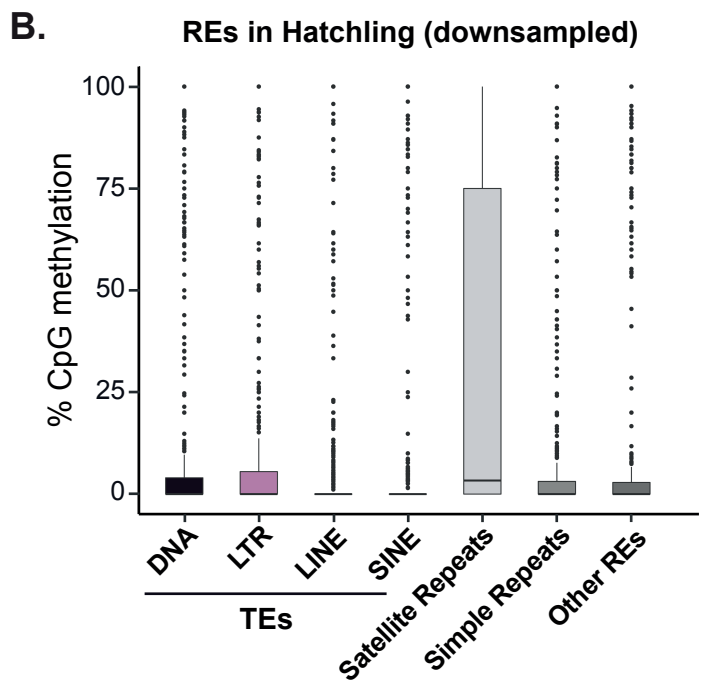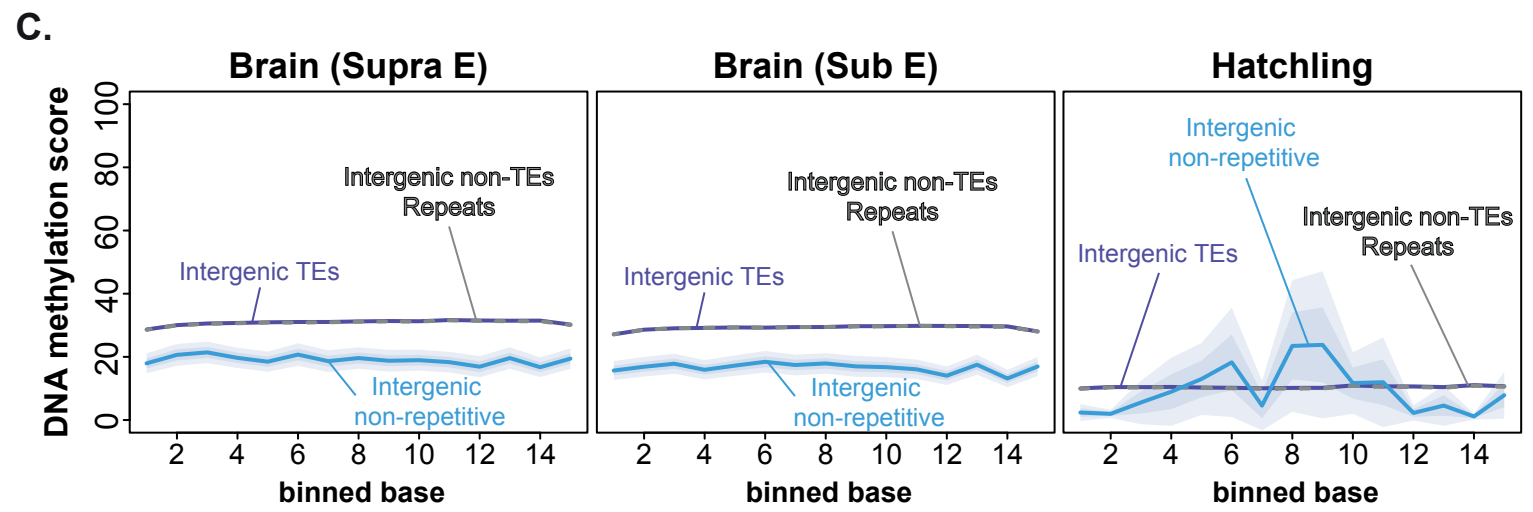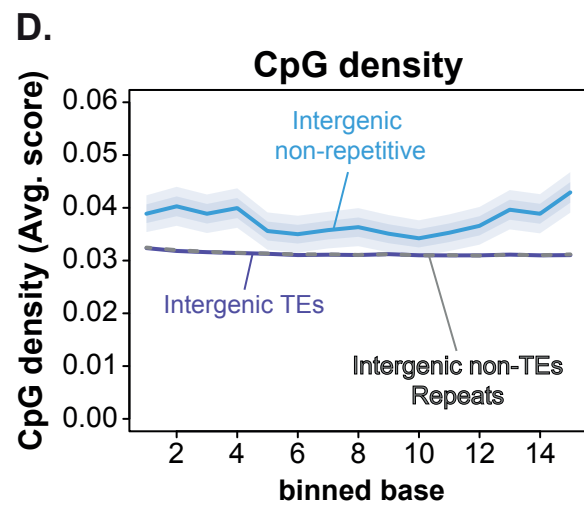

Supplement: Supplementary file 15 — Additional file 15: Figure S9. Pattern of DNA methylation identified by WGBS and RRBS in repetitive elements. A. Box plot describing the percentage of methylation of CpGs contained in repetitive elements (RE) divided by class in the 30 dpf hatchling. B. Box plot depicting the percentage of methylation of CpGs contained in repetitive elements (RE) divided by class in the 30 dpf hatchling and down-sampled based on the number of CpGs in satellite repeats. Box-and-whisker plots have a center line at the median, lower and upper hinges correspond to first and third quartiles, and whiskers extend from hinges to largest or smallest values no further than 1.5 × IQR (inter-quartile range), while data beyond the end of the whiskers are outlying points that are plotted individually. C. Metaplot displays CpG methylation levels of intergenic TEs, intergenic non-transposable repetitive elements and in non-repetitive intergenic regions in Supra E and Sub E brain and hatchling. D. CpG density of the same regions defined in panel C. Each region is divided in 15 bins. [file 12915_2022_1404_MOESM15_ESM.pdf]

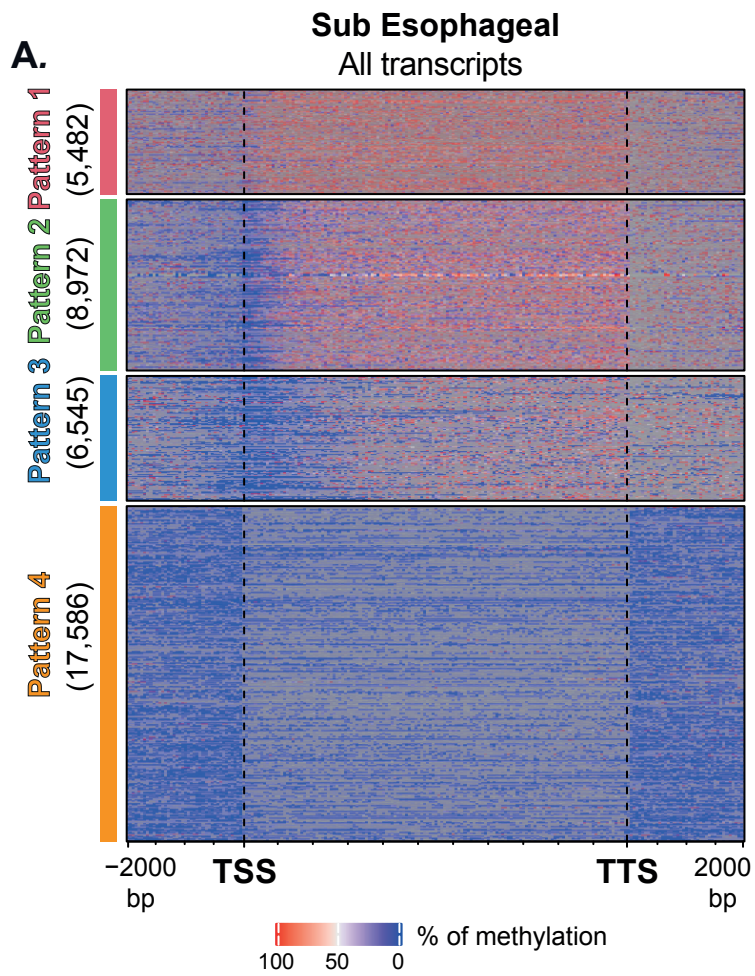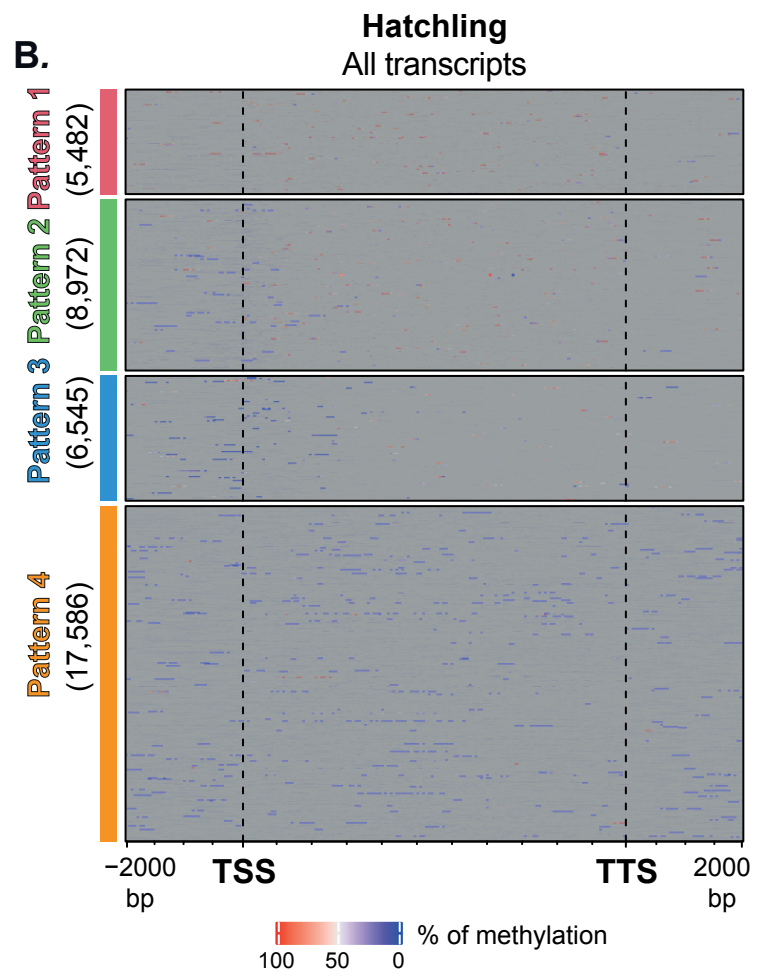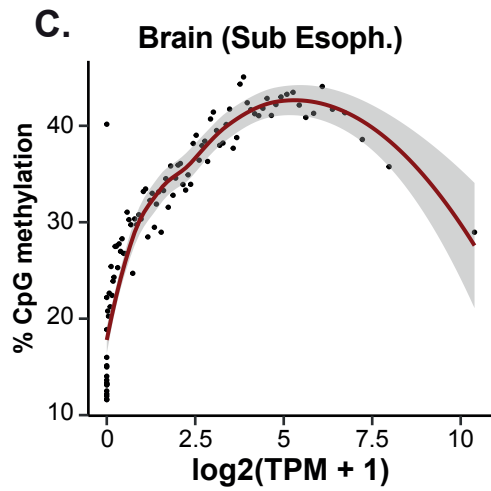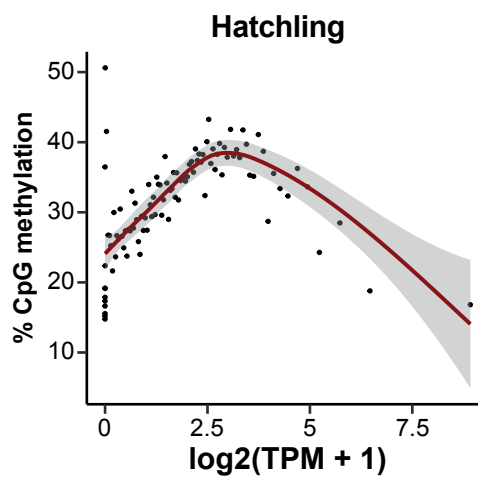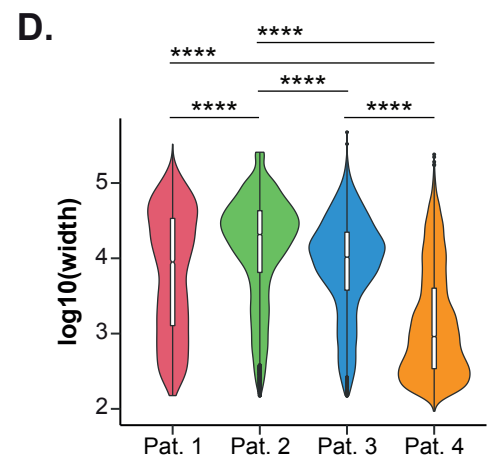

Supplement: Supplementary file 17 — Additional file 17: Figure S10. Relationship between DNA methylation pattern gene expression, and gene length in octopus. A. Heatmap of DNA methylation of all full-length transcripts and 2000 bp upstream and downstream as detected in the Sub E brain WGBS data. Clustering and rank order is dictated by Supra E brain samples shown in Fig. 4A. B. Heatmap of DNA methylation in hatchling full-length transcripts and 2000 bp upstream and downstream. Clustering and rank order is dictated by Supra E brain samples shown in Fig. 4A. C. Overall DNA methylation of transcripts (TSS to TTS of each transcript) in Sub E brain and hatchling is regressed against transcripts expression (log2(TPM+1)). Transcripts were grouped by percentile of expression values and each dot represents the average value of DNA methylation for each percentile. D. Violin plot displays the distribution of transcript length (as log10(width)) in each methylation pattern. Box-and-whisker inside violin plots have a center line at the median, lower and upper hinges correspond to first and third quartiles, and whiskers extend from hinges to largest or smallest values no further than 1.5 × IQR (inter-quartile range), while data beyond the end of the whiskers are outlying points that are plotted individually. p-values were calculated by unpaired non-parametric Kruskal-Wallis test adjusted with Dunn’s multiple comparisons test. **** indicates p-value adjusted < 0.0001. [file 12915_2022_1404_MOESM17_ESM.pdf]

A. EZH2

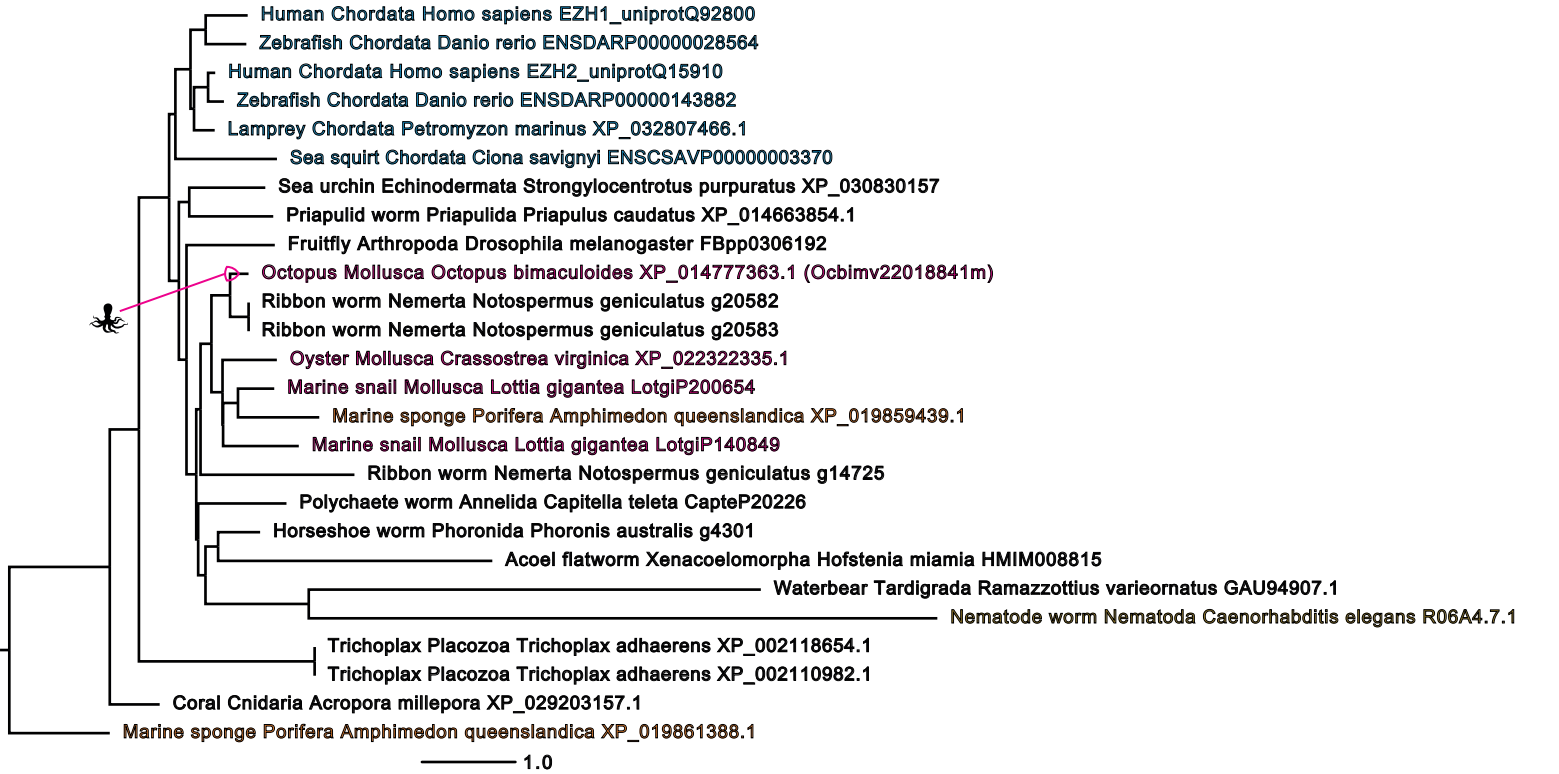

B. KAT2A

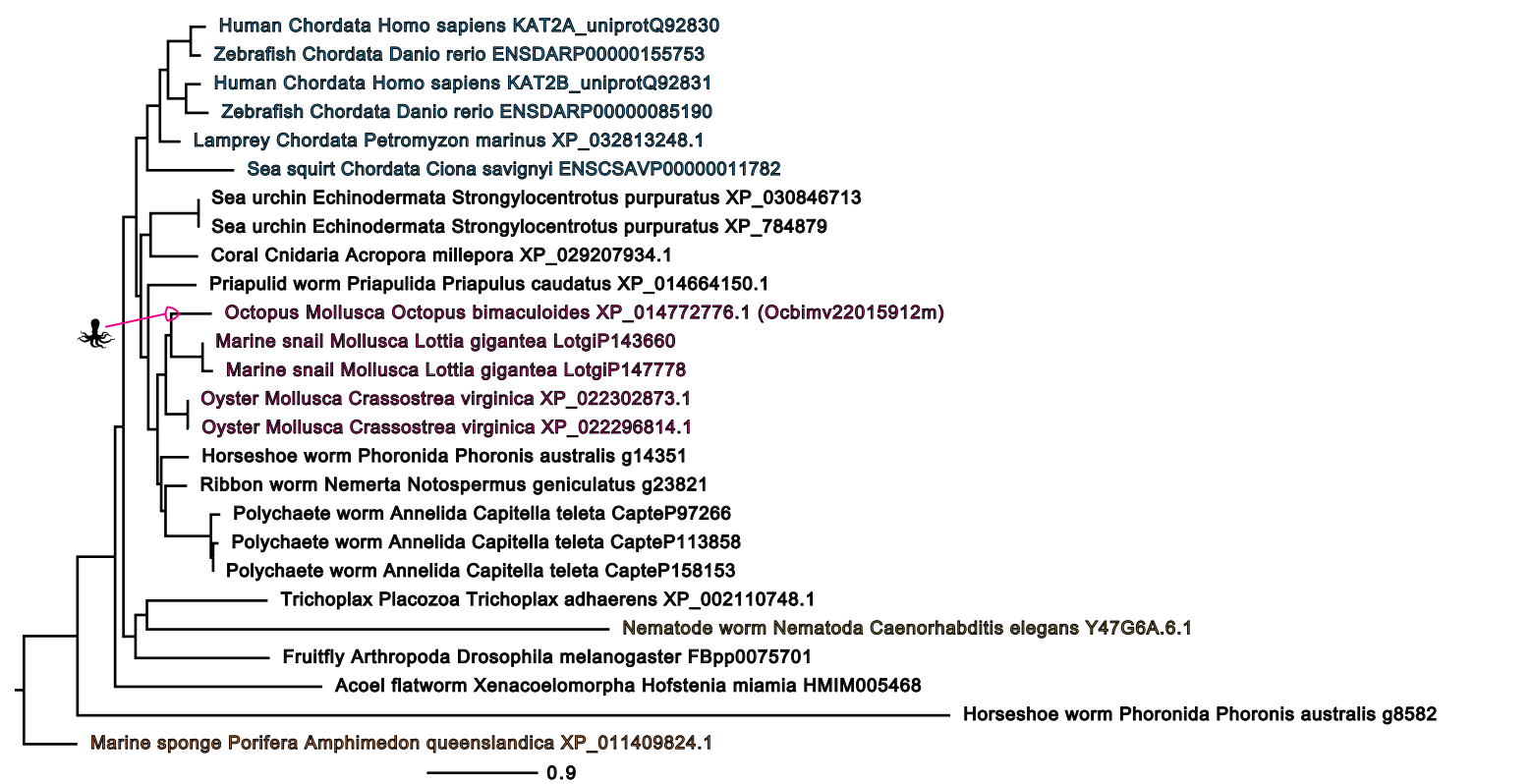

C. HDAC8

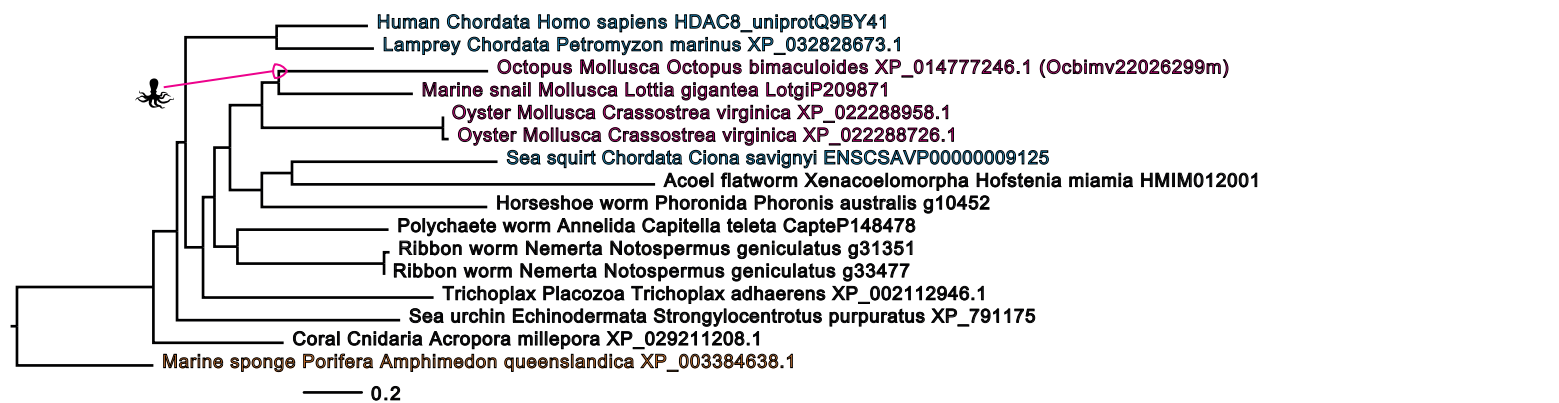

Supplement: Supplementary file 18 — Additional file 18: Figure S11. Supplemental phylogenetic trees. A. Phylogenetic tree of EZH2, responsible of H3K27me3 deposition, in a representative subset of 19 metazoan and outgroup species. B. Phylogenetic tree of KAT2A, mainly responsible of H3K9ac deposition, in a representative subset of 19 metazoan and outgroup species. C. Phylogenetic tree of HADC8, mainly responsible of H3K9ac removal, in a representative subset of 19 metazoan and outgroup species. Colors indicate phyla (blue: Chordata, pink: Mollusca, orange: Porifera; ocra: Nematoda), and octopus are indicated with an icon. [file 12915_2022_1404_MOESM18_ESM.pdf]

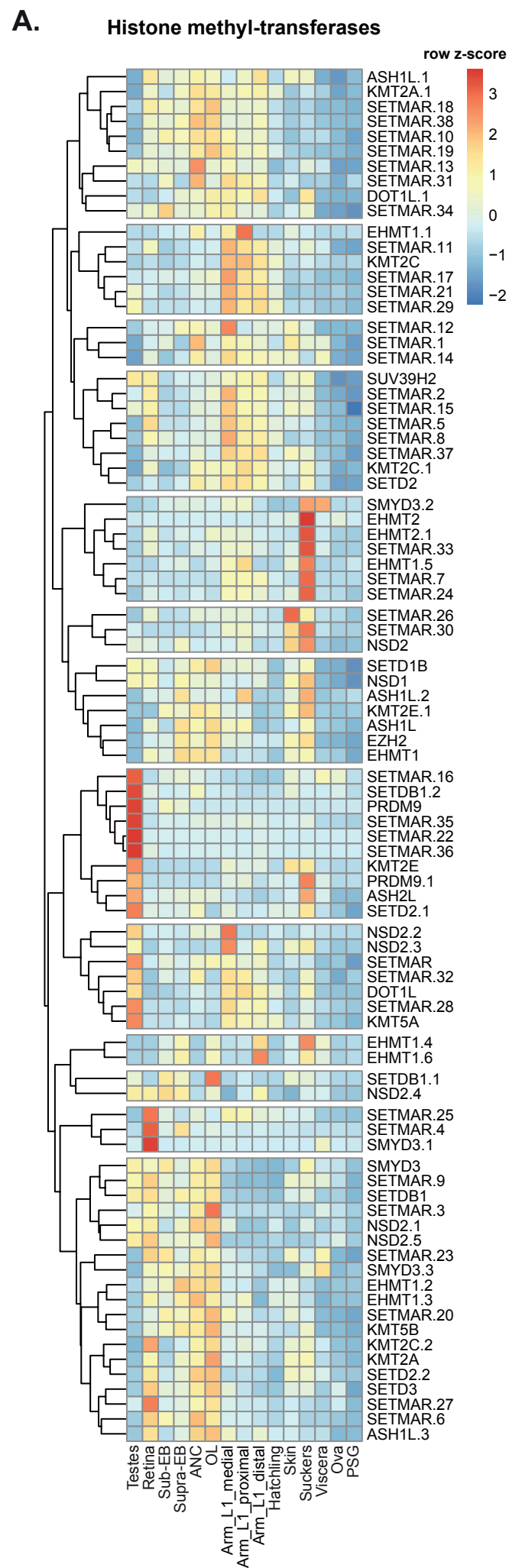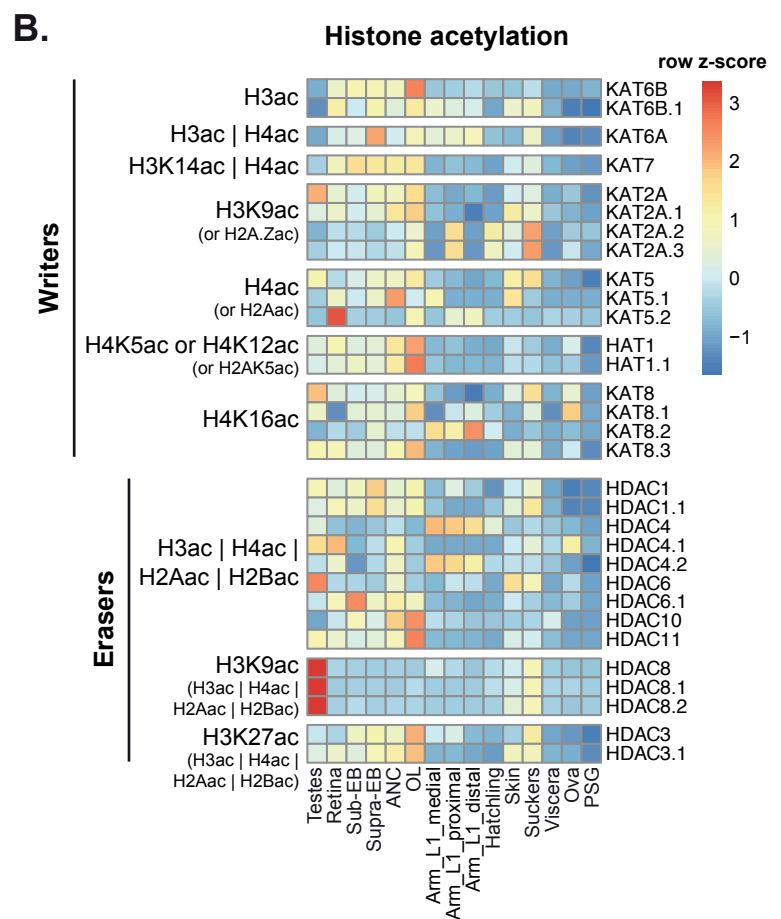

Supplement: Supplementary file 19 — Additional file 19: Figure S12. Histone methyltransferases, acetyltransferases and de-acetylases have a tissue specific expression pattern in octopus. A. Heatmap of the extended panel of histone methyltransferases. B. Heatmap of the main histone acetylation factors, acetyltransferase (writers) and de- acetylases (erasers). [file 12915_2022_1404_MOESM19_ESM.pdf]

A.

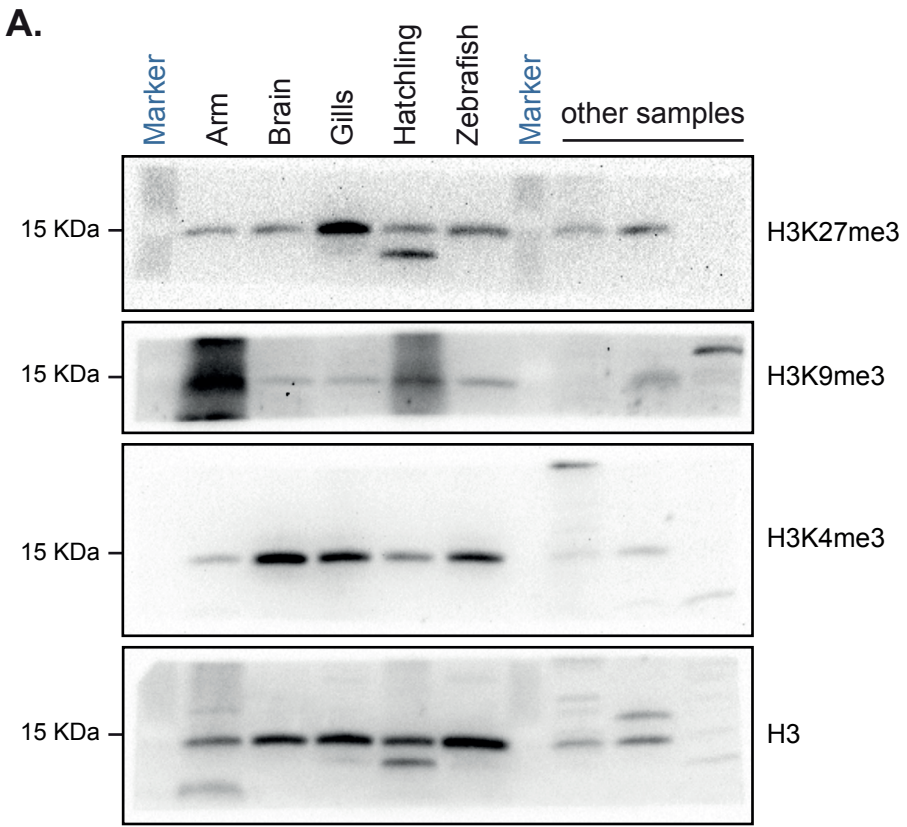

Supplement: Supplementary file 20 — Additional file 20: Figure S13. Original uncropped images of western blot in Fig. 5D. [file 12915_2022_1404_MOESM20_ESM.pdf]

A.

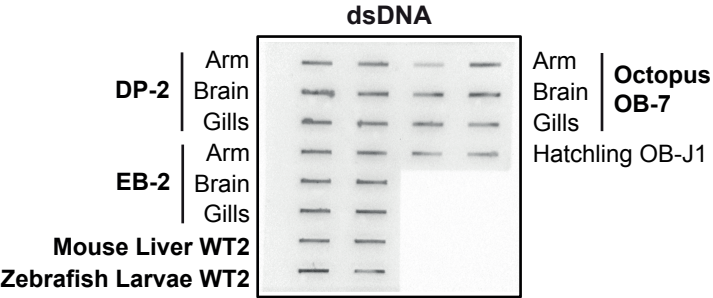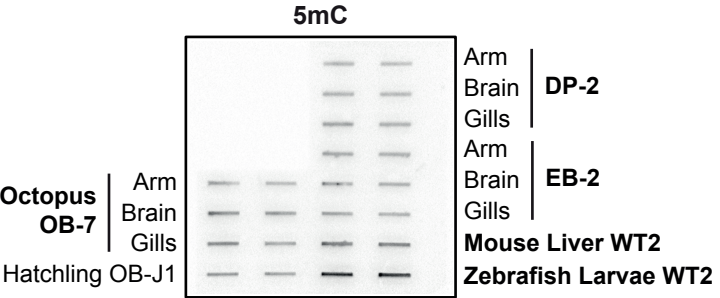

B.

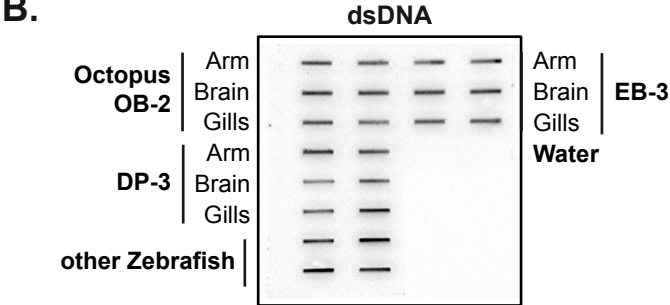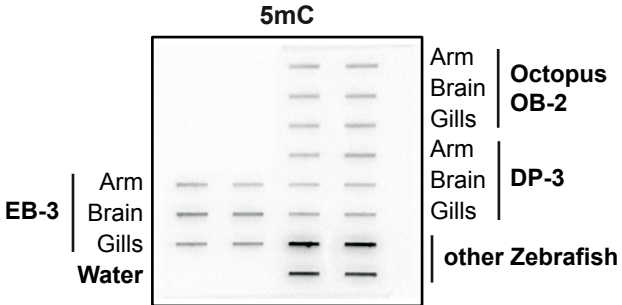

Supplement: Supplementary file 21 — Additional file 21: Figure S14. Original uncropped images of Slot blot in Fig. 6A. A. Blots containing biological replicate 2 and used for quantification. B. Blots containing replicate 3 and run with water used to prepare all samples and solutions. [file 12915_2022_1404_MOESM21_ESM.pdf]

**A.**

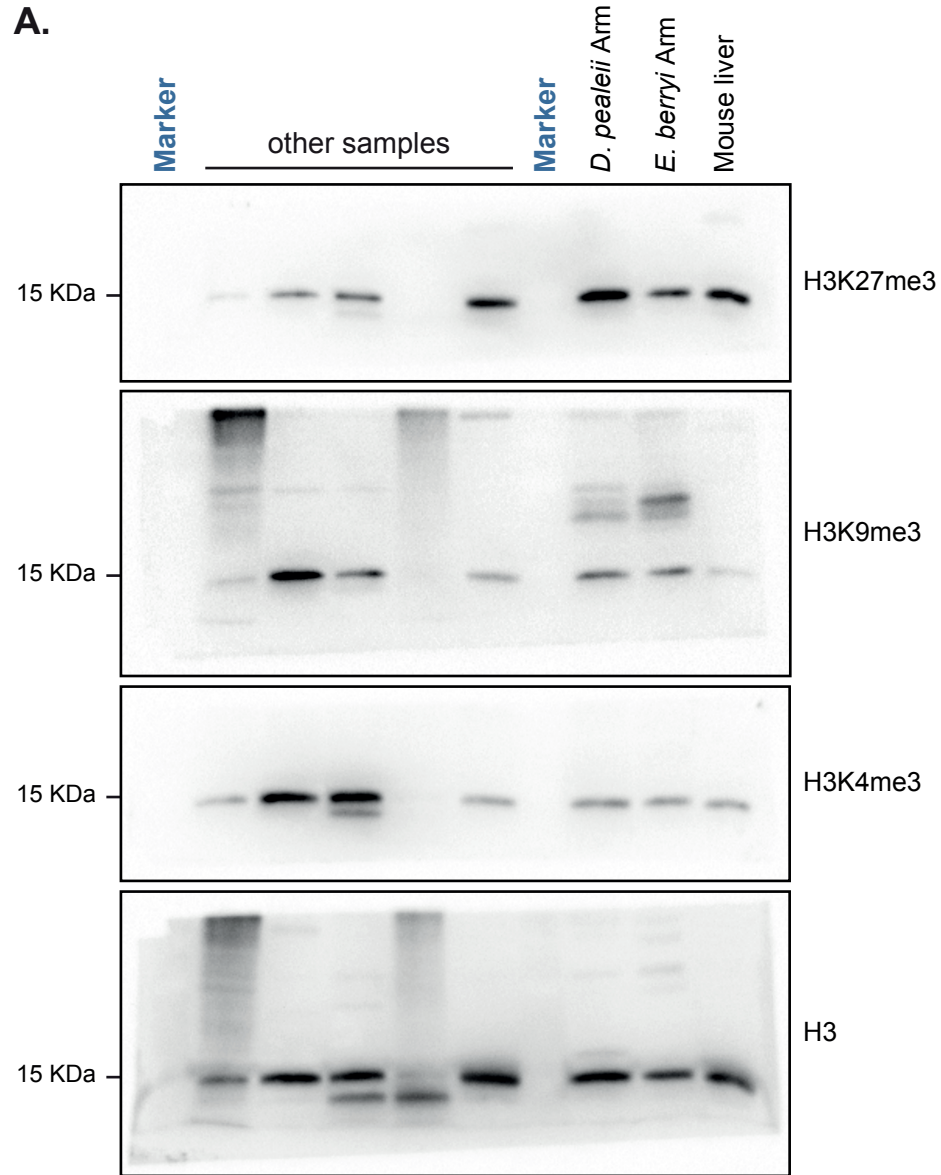

Supplement: Supplementary file 22 — Additional file 22: Figure S15. Original uncropped images of western blot in Fig. 6C. [file 12915_2022_1404_MOESM22_ESM.pdf]
