# Supplementary material for: Epigenetic machinery is functionally conserved in cephalopods
Source: BMC Biol. 2022 Sep 14;20:202. doi: 10.1186/s12915-022-01404-1 (PMC9476566; doi:10.1186/s12915-022-01404-1)

## A. DNMT1_OCTBM

| Domain | Pfam ID | Bit Score | Domain E-values | |
|---|---|---|---|---|
| | | | Ind. | Cond. |
| RFTD | DNMT1-RFD | 138.86 | 1.2e-40 | 2.7e-44 |
| CXXC | zf-CXXC | 47.88 | 1.0e-12 | 2.2e-16 |
| BAH1 | BAH | 55.25 | 5.7e-15 | 1.3e-18 |
| BAH2 | BAH | 55.65 | 4.3e-15 | 9.5e-19 |
| CTD (C-5 cytosine methyltransferase) | DNA_methylase | 165.85 | 1.5e-48 | 3.4e-52 |

HMMER (*O. bimaculoides* protein sequence vs. entire protein sequence database)

## B. DNMT1_OCTBM

| Organism | UniProt ID | Gene ID | % of Identity |
|---|---|---|---|
| Octopus bimaculoides | A0A0L8GEZ1 | *Ocbimv22034501m.g* | 100.00 |
| Danio rerio | Q8QGB8 | *dnmt1* | 56.01 |
| Homo sapiens | P26358 | *DNMT1* | 54.79 |
| Mus musculus | P13864 | *Dnmt1* | 52.24 |

ClustalOmega multiple protein sequence alignment

## C. UHRF1_OCTBM

| Domain | Pfam ID | Bit Score | Domain E-values | |
|---|---|---|---|---|
| | | | Ind. | Cond. |
| UBL | ubiquitin | 52.61 | 2.8e-14 | 6.2e-18 |
| TTD | TTD | 171.29 | 1.3e-50 | 2.9e-54 |
| PHD | PHD | 47.58 | 1.1e-12 | 2.5e-16 |
| SRA | SAD_SRA | 190.13 | 1.9e-56 | 4.2e-60 |
| RING | Not unique ID | Manually annotated | % identity (count) = 64.1% (39) | |

HMMER (*O. bimaculoides* protein sequence vs. entire protein sequence database)

## D. UHRF1_OCTBM

| Organism | UniProt ID | Gene ID | % of Identity |
|---|---|---|---|
| Octopus bimaculoides | A0A0L8IC59 | *Ocbimv22021185m.g* | 100.00 |
| Danio rerio | E7EZF3 | *uhrf1* | 56.37 |
| Homo sapiens | Q96T88 | *UHRF1* | 55.88 |
| Mus musculus | Q8VDF2 | *Uhrf1* | 53.55 |
| Homo sapiens | Q96PU4 | *UHRF2* | 50.19 |
| Mus musculus | Q7TMI3 | *Uhrf2* | 50.00 |

ClustalOmega multiple protein sequence alignment

## E. YDG_OCTBM

| Domain | Pfam ID | Bit Score | Domain E-values | |
|---|---|---|---|---|
| | | | Ind. | Cond. |
| SRA | SAD_SRA | 195.46 | 4.3e-58 | 2.4e-62 |

HMMER (*O. bimaculoides* protein sequence vs. entire protein sequence database)

## F. YDG_OCTBM

| Organism | UniProt ID | Gene ID | % of Identity |
|---|---|---|---|
| Octopus bimaculoides | A0A0L8H8G0 | *Ocbimv22020196m.g* | 100.00 |
| Mus musculus | Q8VDF2 | *Uhrf1* | 34.46 |
| Homo sapiens | Q96T88 | *UHRF1* | 33.53 |
| Danio rerio | E7EZF3 | *uhrf1* | 32.42 |
| Mus musculus | Q7TMI3 | *Uhrf2* | 31.69 |
| Homo sapiens | Q96PU4 | *UHRF2* | 31.08 |

ClustalOmega multiple protein sequence alignment

## G.

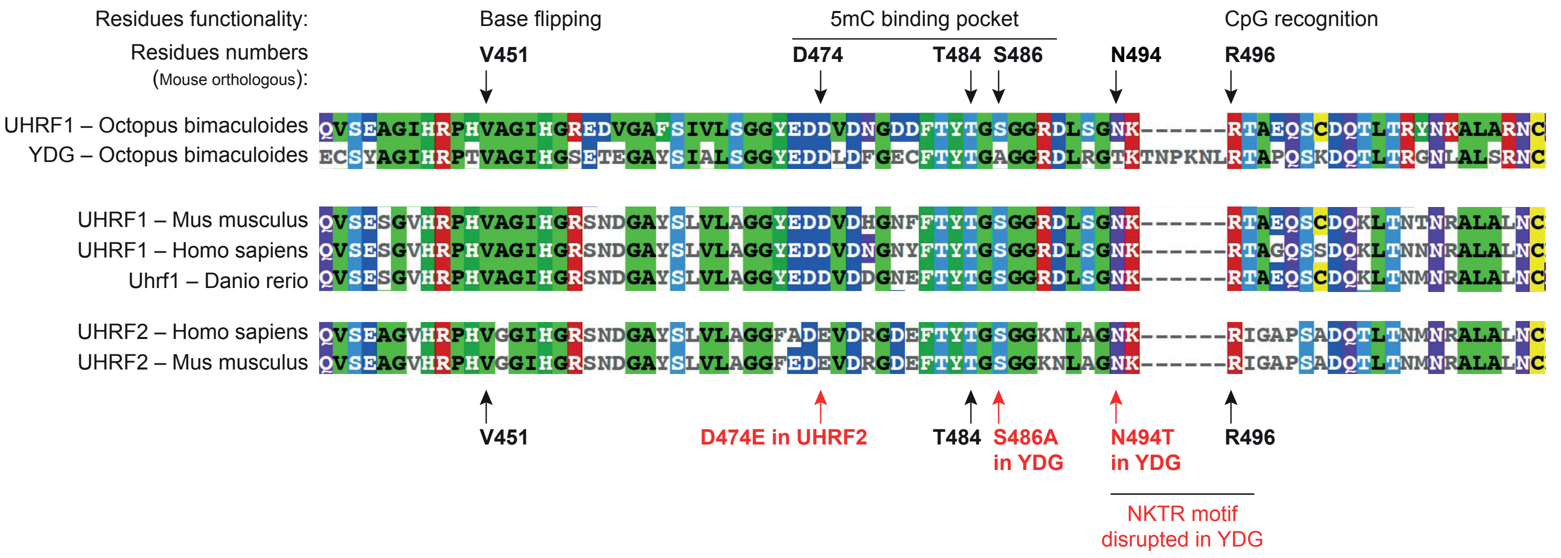

Supplement: Supplementary file 9 — Additional file 9: Figure S5.O. bimaculoides genome encodes conserved features of DNMT1 and UHRF1 but not UHRF2. A. Similarity for each domain of O. bimaculoides DNMT1 determined by HMMER. Bit score indicates homology score. B. Table shows percentage of sequence identity calculated by ClustalOmega for the O. bimaculoides DNMT1 protein compared to human, mouse, and zebrafish in a multiple sequence alignment. C. Table shows similarity for each domain (with a PFAM ID) of O. bimaculoides UHRF1 when compared to the proteome database using HMMER. Bit score indicates homology score. D. Table shows percentage of sequence identity calculated by ClustalOmega for the O. bimaculoides UHRF1 protein compared to human, mouse, and zebrafish in a multiple sequence alignment. E. Similarity for each domain of the O. bimaculoides YDG_OCTBM protein determined using HMMER. Bit score indicates homology score. F. Percent identity calculated by ClustalOmega for the O. bimaculoides YDG_OCTBM protein compared to human, mouse, and zebrafish in a multiple sequence alignment. G. Alignment of the SRA domain in O. bimaculoides YDG_OCTBM to human, mouse, and zebrafish shows that all the major residues needed for the correct functionality of UHRF1 are not conserved in YDG_OCTBM of O. bimaculoides. Alignment to UHRF2 shows no conservation of critical residues between YDG_OCTBM in O. bimaculoides and UHRF2 in human, mouse. Residues functionality is based on mouse orthologs. [file 12915_2022_1404_MOESM9_ESM.pdf]
