# Supplementary material for: Epigenetic machinery is functionally conserved in cephalopods
Source: BMC Biol. 2022 Sep 14;20:202. doi: 10.1186/s12915-022-01404-1 (PMC9476566; doi:10.1186/s12915-022-01404-1)
Supplement: Supplementary file 10 — Additional file 10: Figure S6. Structural modelling of DNMT1 and UHRF1. A. 3D structure of the BAH1, BAH2 and CTD domains of DNMT1 in M. musculus. B. 3D model of BAH1, BAH2 and CTD domains of DNMT1_OCTBM in O. bimaculoides. C. 3D structure of SRA domain of UHRF1 in M. musculus. D. 3D model of SRA domain of UHRF1_OCTBM in O. bimaculoides. [file 12915_2022_1404_MOESM10_ESM.pdf]

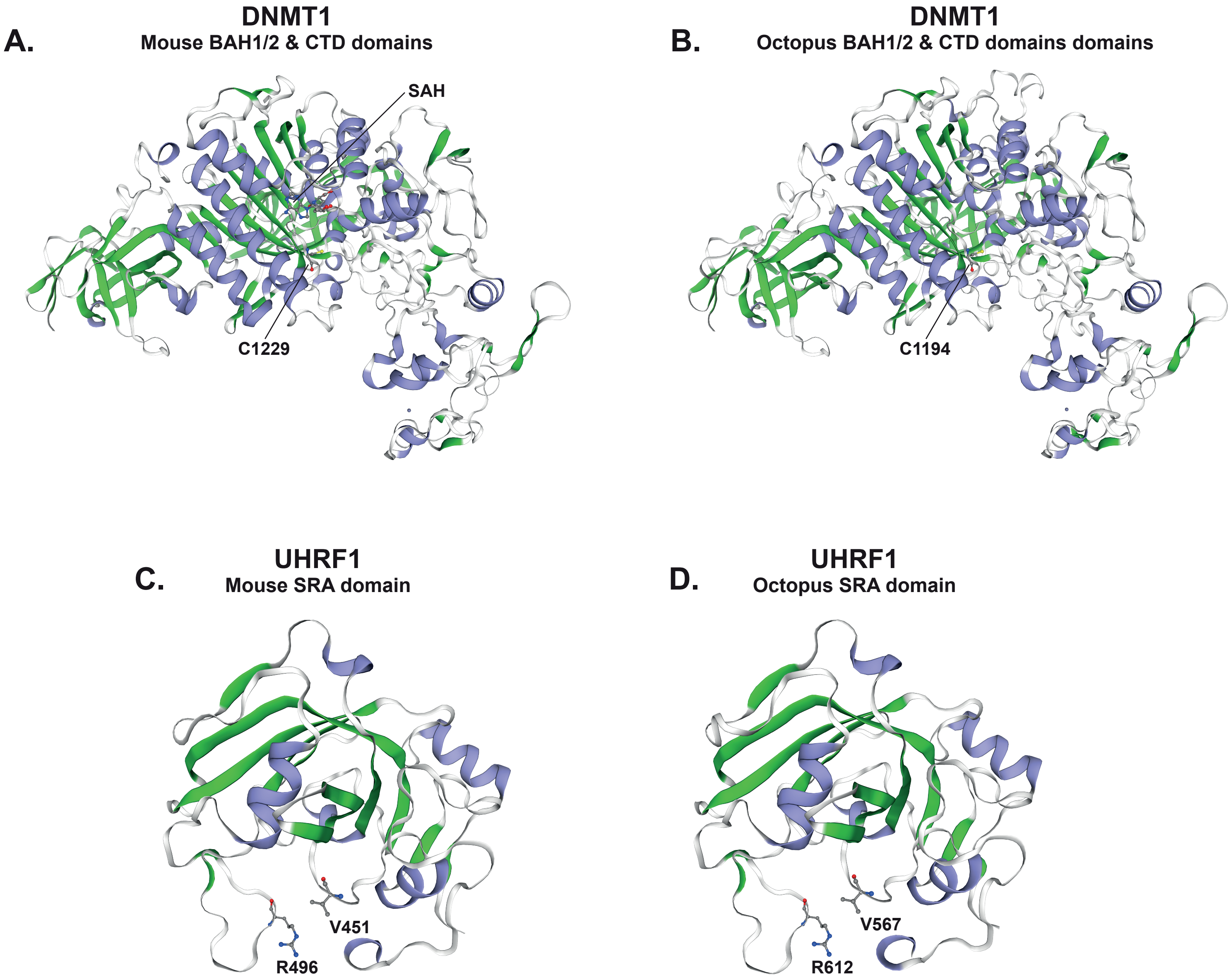
A.
DNMT1
Mouse BAH1/2 & CTD domains
SAH
C1229
B.
DNMT1
Octopus BAH1/2 & CTD domains domains
C1194
C.
UHRF1
Mouse SRA domain
V451
R496
D.
UHRF1
Octopus SRA domain
V567
R612
