# Supplementary material for: Epigenetic machinery is functionally conserved in cephalopods
Source: BMC Biol. 2022 Sep 14;20:202. doi: 10.1186/s12915-022-01404-1 (PMC9476566; doi:10.1186/s12915-022-01404-1)

A.

Total CpGs	80,660,576		
**Sample**	**Covered CpGs**	**Methylated CpGs (>80%)**	**Unmethylated CpGs (<20%)**
**RRBS Hatchling**	2,403,266	87,223	2,172,200
Percent of total	2.98%	3.63%	90.39%
**WGBS Supra E**	64,845,686	3,897,277	55,918,461
Percent of total	80.39%	6.01%	86.23%
**WGBS Sub E**	47,053,504	3,542,069	40,626,272
Percent of total	58.34%	7.53%	86.34%

B.

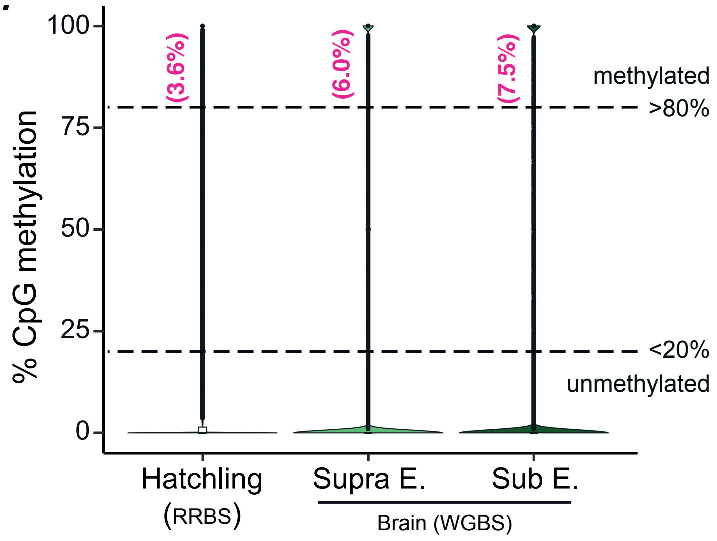

C.

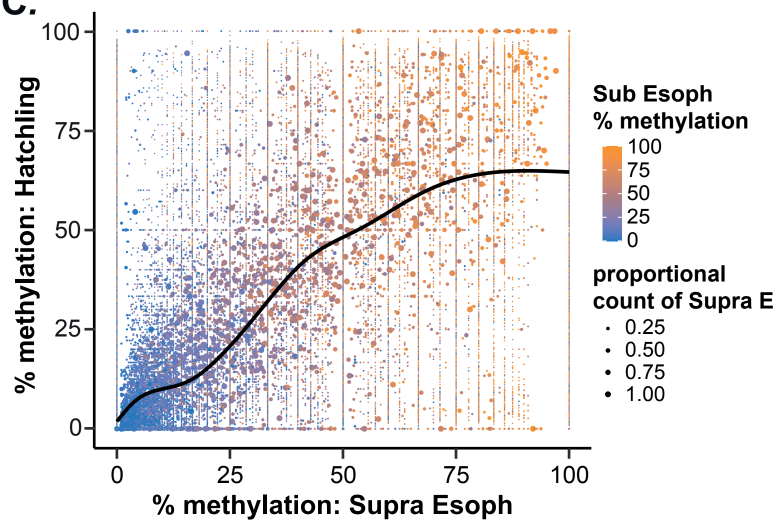

D.

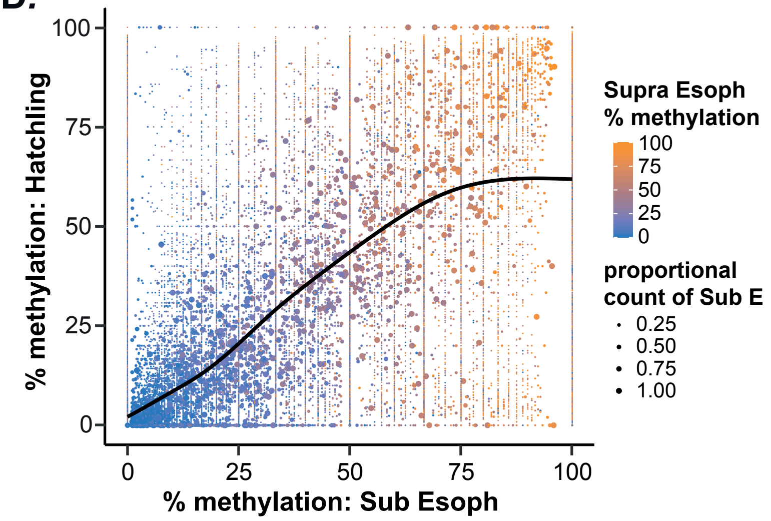

Supplement: Supplementary file 14 — Additional file 14: Figure S8. Pattern of DNA methylation identified by WGBS and RRBS in octopus tissues. A. Table describing the number and relative percentage of CpGs covered in the O. bimaculoides genome by each technique and sample analyzed. CpGs were classified based on the percentage of methylation as methylated (> 80%) or not methylated (< 20%). B. Scaled violin plot of CpGs identified by WGBS and RRBS in Supra E and Sub E brain and in one whole-body 30 dpf hatchling. Box-and-whisker inside violin plots have a center line at the median, lower and upper hinges correspond to first and third quartiles, and whiskers extend from hinges to largest or smallest values no further than 1.5 × IQR (inter-quartile range), while data beyond the end of the whiskers are outlying points that are plotted individually. Numbers on the lines indicate the percent of CpGs that are detected as >80% methylated. C. Scatter plot of DNA methylation levels of common CpGs across Supra Esophageal (Supra E) and Hatchlings. Dot color represents DNA methylation levels in Sub Esophageal (Sub E) and size of the dots indicates scaled proportion of CpGs represented by each dot. D. Scatter plot of DNA methylation levels of common CpGs across Sub Esophageal (Sub E) and Hatchlings. Dot color represents DNA methylation levels in Supra Esophageal (Supra E) and size of the dots indicates scaled proportion of CpGs represented by each dot. [file 12915_2022_1404_MOESM14_ESM.pdf]
